# Solvation Shifts the Band-Edge Position of Colloidal Quantum Dots by Nearly 1 eV

**DOI:** 10.1021/jacs.4c00402

**Published:** 2024-03-26

**Authors:** Yan B. Vogel, Le Nhan Pham, Maarten Stam, Reinout F. Ubbink, Michelle L. Coote, Arjan J. Houtepen

**Affiliations:** † Department of Chemical Engineering, Delft University of Technology, Van der Maasweg 9, 2629 HZ Delft, The Netherlands; ‡ Institute for Nanoscale Science and Technology, College of Science and Engineering, Flinders University, Bedford Park, South Australia 5042, Australia

## Abstract

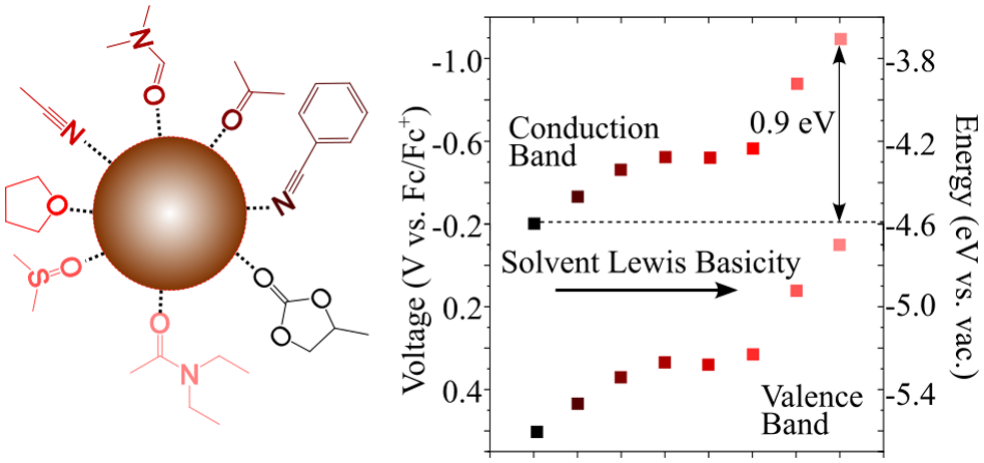

The optoelectronic properties of colloidal quantum dots (cQDs) depend critically on the absolute energy of the conduction and valence band edges. It is well known these band-edge energies are sensitive to the ligands on the cQD surface, but it is much less clear how they depend on other experimental conditions, like solvation. Here, we experimentally determine the band-edge positions of thin films of PbS and ZnO cQDs via spectroelectrochemical measurements. To achieve this, we first carefully evaluate and optimize the electrochemical injection of electrons and holes into PbS cQDs. This results in electrochemically fully reversible electron injection with >8 electrons per PbS cQDs, allowing the quantitative determination of the conduction band energy for PbS cQDs with various diameters and surface compositions. Surprisingly, we find that the band-edge energies shift by nearly 1 eV in the presence of different solvents, a result that also holds true for ZnO cQDs. We argue that complexation and partial charge transfer between solvent and surface ions are responsible for this large effect of the solvent on the band-edge energy. The trend in the energy shift matches the results of density functional theory (DFT) calculations in explicit solvents and scales with the energy of complexation between surface cations and solvents. As a first approximation, the solvent Lewis basicity can be used as a good descriptor to predict the shift of the conduction and valence band edges of solvated cQDs.

## Introduction

Colloidal quantum dots (cQDs) are of interest in various scientific and technological disciplines due to their tunable optoelectronic properties.^1^ Their absorption and emission color can be varied by changing the composition and size to cover a wide wavelength range, and the band alignment in cQD devices can be tuned by modifying the absolute band-edge energies by changing the ligands passivating the cQDs.^2,3^ The size-tunable emission wavelength has led to the commercialization of cQD technology as emitters in liquid crystal display backlights,^4^ and the band-edge shift with ligands has facilitated the development of solar cells^2^ and light-emitting diodes.^5^

The absolute energy levels of cQDs are generally measured by photoelectron spectroscopies in vacuum.^6^ Yet, in many cases, cQDs are dispersed in a solvent. Examples of this range from fundamental studies of charge dynamics^7−9^ to applications of cQDs in photocatalysis,^10^ doping engineering,^11,12^ bioimaging,^13^ and light-emitting electrochemical cells.^14^ In such cases, the assumption is often that the absolute energy levels of cQDs are the same as those in a vacuum. However, this has not been experimentally tested. In addition, the sensitivity of the band positions to surface ligands suggests that the environment can have a significant effect.

We measured the conduction band position of PbS and ZnO cQDs films of a range of sizes using spectroelectrochemistry and found that it varies by nearly 1 eV in different solvents without affecting the bandgap energy. This shift is observed in cQDs made of different materials and different sizes and passivated with different ligands. This advancement was made possible by accomplishing stable electrochemical n-type doping of PbS cQDs. Density functional theory (DFT) calculations of a PbS cQD model in five explicit solvents reproduce the experimental trends in the energy level shift and give insight into the molecular origin of this shift. The magnitude of the band-edge shift correlates with the solvent-cQD complexation energy and can be understood by charge rearrangement between the solvent and cQD surface. These findings will guide fundamental studies and the development of technologies where cQDs are dispersed in a solvent or embedded in an environment where coordination to the surface may occur such as a polymer matrix.

## Results and Discussion

### Spectroelectrochemical Determination of the Stability Limits of PbS cQDs

**Figure 1:**
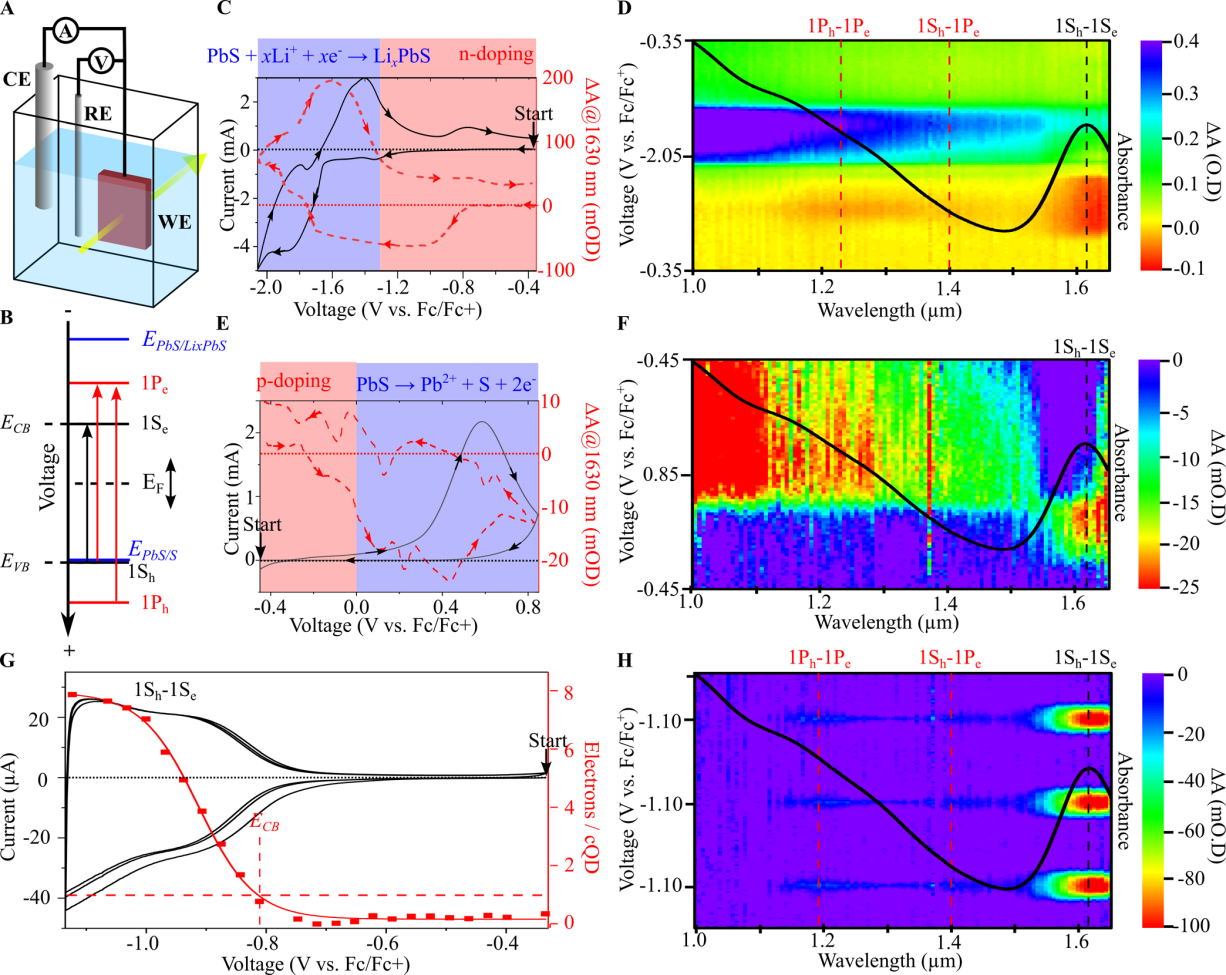
Spectroelectrochemistry of PbS cQDs. (A) Schematics of the spectroelectrochemical setup and (B) band diagram of the PbS cQD. The band diagram is constructed based on the spectroelectrochemical data from (C−H). (C, E) Cyclic voltammograms (black solid line) and Δ*A* vs voltage at 1630 nm (red dashed line) and (D, F) voltage–wavelength−Δ*A* plots of PbS cQDs films. (G) Cyclic voltammetry (black line), number of electrons per cQD (symbols and red line), and (H) voltage–wavelength−Δ*A* plot of PbS cQDs within the stability region. The black line in (D, F, H) is an absorption spectrum of a PbS cQD dispersion in tetrachloroethylene. The dotted lines in (C, E, G) area guide to the eye for a zero current (black) and Δ*A* (red), and the measurement starting potential is indicated with an arrow and corresponds to the OCP. All spectroelectrochemical measurements were performed in acetonitrile in 0.1 M LiClO_4_ under inert atmosphere and at a scan rate of 50 mV/s. The PbS cQDs have a diameter of 5.5 nm and a bandgap of 0.77 eV and are capped with ethanedithiol ligands.

We used a spectroelectrochemical approach to access the conduction/valence band (CB/VB) energy levels (*E*_CB_/*E*_VB_) of films of cQDs immersed in different solvents. A scheme of the setup is shown in Figure 1A. It consists of a three-electrode electrochemical cell operating in a potentiostatic mode coupled to a spectrophotometer. A cQD film on an indium tin oxide on glass substrate is used as the working electrode (WE), a silver wire as the quasi-reference electrode (RE), and a platinum sheet as the counter electrode (CE). These electrodes are immersed in an electrolyte solution of a solvent (e.g., acetonitrile) with an ionic salt (LiClO_4_, unless otherwise stated). Hence, the cQDs are solvated. The cQD synthetic procedure and film assembly are described in the Methods section. A potential is applied between WE and RE, calibrated with respect to the ferrocene/ferrocenium redox couple (Fc/Fc^+^), and the current passing between WE and CE is measured. At the same time, the cQD film differential absorbance (Δ*A*, the difference in absorbance between open circuit and the applied potential) is measured through the WE. By changing the potential with respect to its initial value (open-circuit potential, OCP), the Fermi level (*E*_F_) is shifted, as schematically shown in the energy diagram of Figure 1B.^15,16^ Decreasing the potential to values below *E*_CB_ should lead to electron population into the CB (electrochemical n-type doping), while increasing the potential to values above *E*_VB_ should lead to hole population into the VB (electrochemical p-type doping). In both cases, this should result in an absolute increase in current^9^ and a decrease in absorbance due to Pauli blocking.^17^

We first investigate the electrochemical stability limits of films of PbS cQDs capped with ethanedithiol ligands (PbS-EDT hereafter) with a diameter of 5.5 nm and a bandgap of 0.77 eV (1S exciton peak at 1630 nm) using spectroelectrochemistry in a wide potential range, as shown in Figure 1C–F. We apply a voltage ramp while simultaneously measuring the resulting current (cyclic voltammetry) and the Δ*A* spectra. We performed experiments at potentials below (Figure 1C,D) and above (Figure 1E,F) the OCP to explore both electron and hole injection, respectively.

For electron injection (Figure 1C,D), the experiment starts at the OCP, and the potential is decreased to −2.05 V vs Fc/Fc^+^ and swept back to the OCP. As the potential is lowered, the Δ*A* spectra first show a decrease in absorbance (bleach) starting at ca. −0.8 V vs Fc/Fc^+^. The absorption bleach is centered at 1630 nm, which corresponds to the 1S_h_1S_e_ transition in the steady-state absorption spectrum (Figure 1D, black solid line).^18,19^ We attribute the bleach to Pauli blocking due to electron population into the 1S_e_ levels, as previously shown with different cQD materials.^11,20−24^

When the potential is made more negative two additional bleaches centered at 1220 and 1400 nm appear at ca. −1.0 V vs Fc/Fc^+^ (Figure S1). These features correspond to the 1P_h_-1P_e_ and 1S_h_1P_e_ transitions, respectively (see Figure S1 for details). The bleach saturation of the 1S_h_-1S_e_ transition and the appearance of the 1P_h_-1P_e_ bleach demonstrate complete filling of the 1S_e_ level with eight electrons based on the 8-fold degeneracy in PbS cQDs^25,26^ (Figure 1C, red shaded area).

However, the potential is lowered below −1.2 V vs Fc/Fc^+^, there is an increase in current and in Δ*A* over the whole spectrum that is absent in a blank indium tin oxide electrode over the same potential range (Figures 1D and S2). We attribute the increase in Δ*A* to free carrier absorption or enhanced light scattering in heavily doped PbS cQDs. Cyclic voltammetry at increasingly negative potential (Figure S3) shows a transition from a reversible to a quasi-reversible behavior occurring at −1.35 V vs Fc/Fc^+^. To investigate the origins of this transition, we performed ex situ X-ray photoelectron spectroscopy (XPS) on the reduced films (Figure S4). The XPS data show the presence of Li in the reduced samples, even after thoroughly washing to remove the excess electrolyte. This suggests the formation of Li_*x*_PbS. We hypothesize that this occurs by lithium intercalation
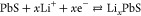


Lithium intercalation in metal sulfides, such as CoS_2_, MoS_2_, and VS_4_, is well known.^27^ We note that the integrated cathodic and anodic currents are of similar magnitude, indicating that the above reaction is chemically reversible, but given the large overpotential between the anodic and cathodic peak positions, the reaction is electrochemically quasi-reversible.^28^ This is typical for lithiation reactions.^27^ However, permanent, irreversible changes occur in the absorption spectrum, leaving an overall increase in absorption at all wavelengths at the end of the cyclic scan (Figure 1C,D). We suspect that the lithiation and delithiation reactions cause irreversible changes to the QD structure, such as a change in surface composition.

To explore hole injection, we sweep the potential from the OCP to +0.85 V vs Fc/Fc^+^ and back to the OCP (Figure 1E,F). This results in an initial increase in current and a decrease in ΔA at the 1S_h_-1S_e_ transition, proving hole injection into the 1S_h_ level. For potentials above 0.0 V vs Fc/Fc^+^, we observe an overall decrease in the absorption spectrum (Figures 1F and S5), which we attribute to anodic dissolution, since both Pb^2+^ and S are soluble in acetonitrile
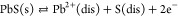


This reaction is evidenced by the irreversible cyclic voltammogram and the decrease in absorbance occurring at all wavelengths. We note that in this case no cathodic wave is detected in the reverse scan, showing that this reaction is also chemically irreversible.

From these measurements we establish a stable electrochemical window between 0.0 and −1.2 V vs Fc/Fc^+^, while the CB and VB positions are found to be around −0.8 V vs Fc/Fc^+^ and 0.0 V vs Fc/Fc^+^, yielding an electrochemical bandgap of 0.8 eV, in line with the optical bandgap of 0.76 eV. We note that this is a rough estimate of the CB and VB positions; in the next section, we will precisely estimate the CB/VB level.

### Reversible Electrochemical n-Doping and Determination of the CB Level

Now that we have identified the electrochemical stability window for PbS cQDs, it becomes possible to reversibly charge and discharge the cQDs. In the rest of the paper, we focus on n-doping because it has a much wider stability window. Figure 1G,H shows the resulting cyclic voltammogram and Δ*A* spectra when staying inside this stable electrochemical window. Note the much smaller current range in this potential range and its capacitive behavior. The reversibility of the cyclic voltammogram and Δ*A* spectra over several scans demonstrates that charges are injected and extracted from the cQDs reversibly. The shape of the cyclic voltammogram follows the characteristic density of states of cQDs.^16^ The ratio between the injected and extracted charge is 90% for the CV shown in Figure 1G, but it increases to 98% by increasing the scan rate (Figure S6). We attribute the lower extracted charges to electrons being consumed by redox impurities such as oxygen and water as demonstrated in our previous work,^29^ which can be avoided by rapidly extracting the electrons with increasing the scan rate. This is a remarkable result because stable electron injection is uncommon and usually limited to few metal oxides.^22,30^

The number of 1S electrons ⟨N_1Se_⟩ per cQD can be extracted from the fractional bleach of the 1S_h_1S_e_ transition via ⟨N_1Se_⟩ (V) = g_1Se_Δ*A*(V)/*A*_0_, where *A*_0_ is the ground state absorption and g_1Se_ is the degeneracy of the 1S electron level, i.e. g_1Se_ = 8 for PbS cQDs.^31^ This is shown in Figure 1G as the red data points. To the plot of ⟨N_1Se_⟩(V) we fitted an error function (red solid line), i.e., the integral of the Gaussian density of states of the 1S_e_ level. ⟨N_1Se_⟩ levels off as the 1S_e_ level is completely populated and the 1P_e_ level starts to be filled. The first derivative of this error function gives a Gaussian curve with a full width at half-maximum (fwhm) of 150 meV. However, this value is subject to thermal population broadening. As the Fermi level in the cQDs is raised with the applied potential (*V*), the 1S_e_ level is filled up with electrons following a Fermi–Dirac distribution, following eq 1
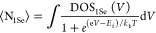
1

Evaluated at 300 K, a measured fwhm of 150 meV corresponds to a DOS function with a fwhm of 109 meV (see Figure S7). The fwhm of the 1S absorption peak is 90 meV, quite close to the extracted DOS of the 1S_e_ level, confirming that the electrochemical injection of 1S_e_ electrons is reversible, as it does not involve significant overpotentials.

To quantify the conduction band level *E*_CB_ in solvated cQDs, we define it as the potential required to inject one electron into the 1S_e_ level, giving an absorption bleach that is 12.5% of the maximum (Figure 1G, red dashed lines).

### Size Dependence of the CB Level

**Figure 2:**
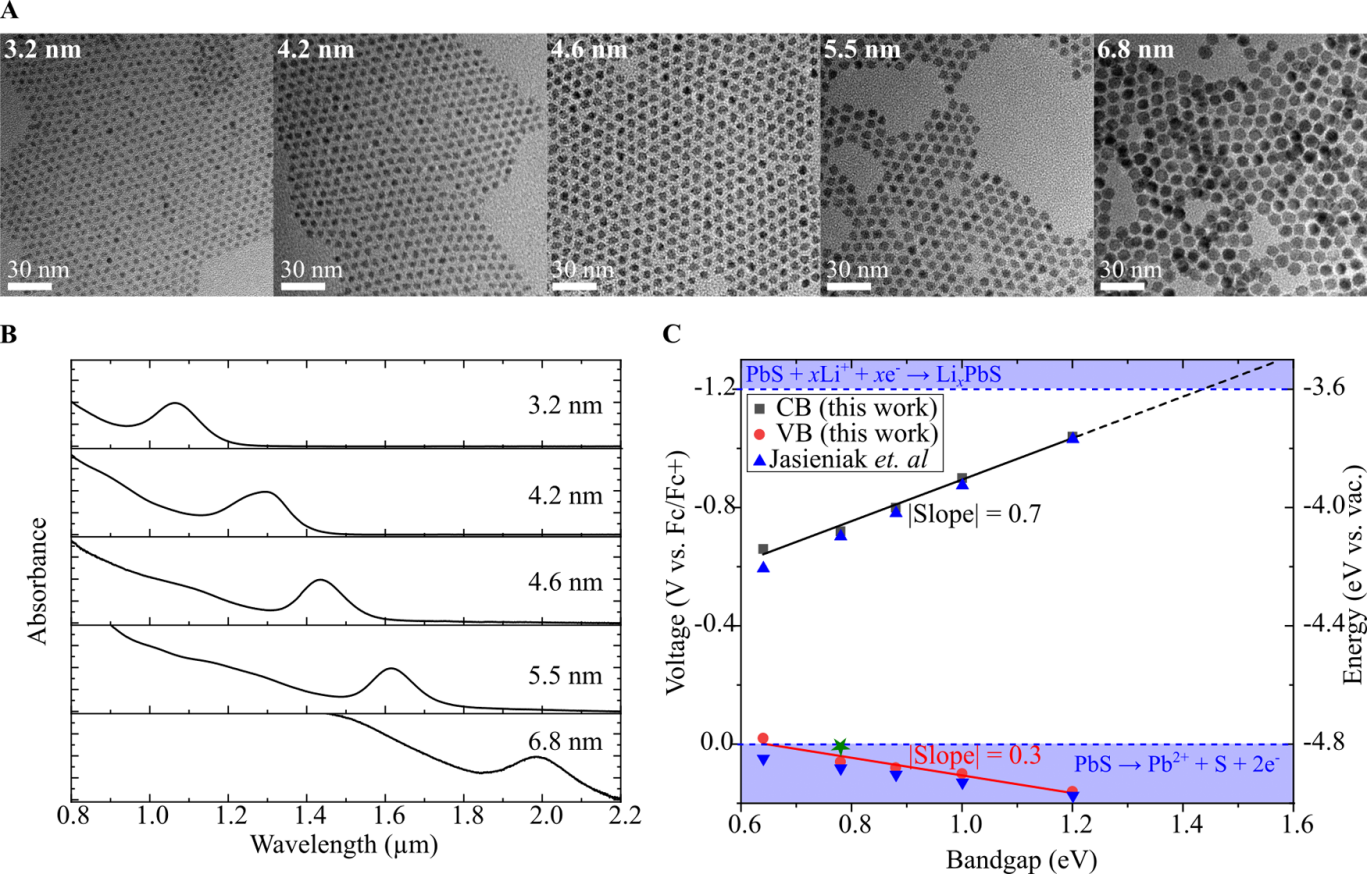
Size dependence of the band-edge positions of PbS cQDs. (A) Transmission electron microscopy images and (B) absorbance spectra in tetrachloroethylene of PbS cQDs of different sizes. The scale bar is 30 nm, and the particle diameter is shown as an inset. (C) Conduction and valence band-edge energies of PbS cQDs as a function of the bandgap as obtained in this work (black squared and red circles, respectively) and from Jasieniak et al.’s work (blue triangles). Note that the data from this work is for ethanedithiol-capped PbS cQDs and that of Jasieniak et. al is for oleate-capped PbS cQDs. In this work, *E*_CB_ was determined by spectroelectrochemistry and *E*_VB_ was inferred by adding the optical bandgap to this value. The green star shows *E*_VB_ as estimated by spectroelectrochemistry from Figure 1E. The blue shaded areas indicate the voltage region where other electrochemical processes occur. All spectroelectrochemical measurements were performed in acetonitrile in 0.1 M LiClO_4_ under inert atmosphere conditions.

In the previous section, we demonstrated an experimental approach to determine *E*_CB_ of solvated cQDs. In this section, we test the validity of this approach by determining the size dependence of *E*_CB_. We varied the cQD diameter by adjusting the synthesis temperature and precursor ratio (see Figure 2A and the Methods section) and determined their bandgap by taking the 1S_h_1S_e_ excitonic peak energy from steady-state absorption spectroscopy (Figure 2B). The values of *E*_CB_ were determined for all sizes using spectroelectrochemistry with 0.1 M LiClO_4_ in acetonitrile as electrolyte solution, see Figures S8–S10. The resulting relation between *E*_CB_ and bandgap is shown in Figure 2C (black squares). *E*_VB_ is also shown in Figure 2C (red circles) and was obtained by subtracting the optical bandgap from *E*_CB_. *E*_VB_ as estimated by spectroelectrochemistry from Figure 1E is also shown in Figure 2C (green star). The obtained values are surprisingly similar in absolute value from those obtained by Jasieniak et al. by UPS (Figure 2C, blue triangles),^32^ despite the difference in ligands (ethanedithiol vs oleate) which should shift the band edges by several hundreds of meV.^2,3^ This fact already hints at an underlying effect that could be attributed to the difference in environment (acetonitrile vs vacuum). Linear fitting of the obtained *E*_CB_ and *E*_VB_ as a function of the cQD bandgap in Figure 2C results in a slope of 0.7 for *E*_CB_ and −0.3 for *E*_VB_, which implies that the electron effective mass is smaller than the hole effective mass. This is in contrast to the typical assumption that the electron and hole effective mass are the same in PbS, but in line with the UPS experiments (Figure 2C).^32^ Therefore, we conclude that our approach permits an accurate estimation of the *E*_CB_.

As the cQD size decreases, the bandgap increases and *E*_CB_ shifts to more negative voltages and outside the stable electrochemical window (Figure 2C). We found that the stable potential window does not change significantly with the cQD size (Figure S11), resulting in a crossing of *E*_CB_ outside the stable electrochemical window for PbS cQDs with a bandgap of 1.45 eV, meaning that these (or smaller size) cQDs will not be stable upon electron population into the CB. In agreement with this, we observed no population of the 1S_e_ level for PbS-EDT cQDs with band gaps of 1.6 eV.

From these measurements, we conclude that we developed a reliable method to determine the absolute band-edge positions of solvated cQDs. Next, we proceeded to study the influence of solvation on the absolute band-edge positions.

### Solvent-Dependent Conduction Band Edge

**Figure 3:**
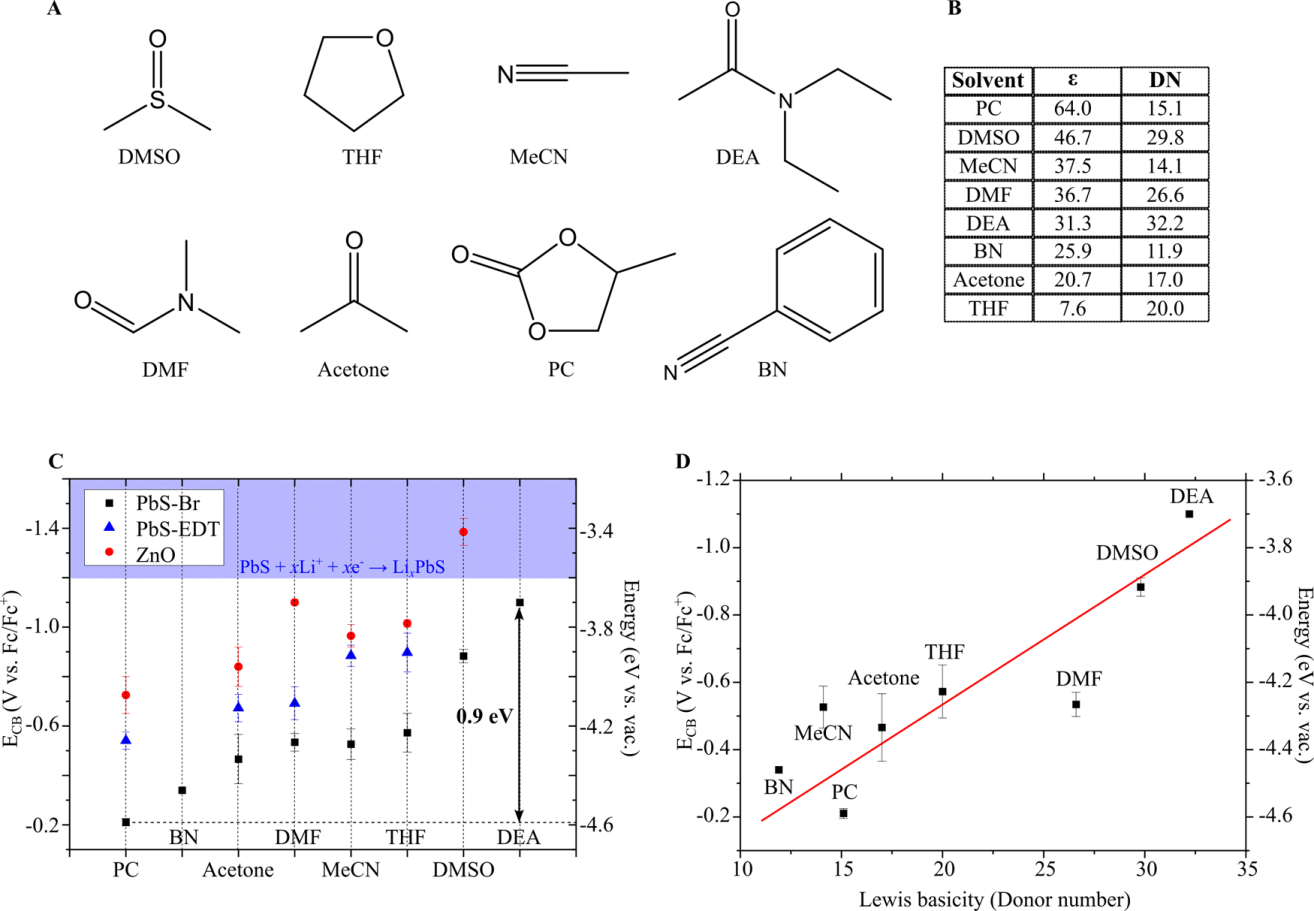
Solvent dependence of cQDs band-edge positions. (A) Solvent structures of dimethyl sulfoxide (DMSO), tetrahydrofuran (THF), acetonitrile (MeCN), dimethylformamide (DMF), acetone, propylene carbonate (PC), benzonitrile (BN), and *N*,*N*-diethylacetamide (DEA). (B) Lewis basicity (DN) and relative permittivity (ε) of the explored solvents. The Gutmann donor number was used as a scale for Lewis basicity. (C) *E*_CB_ of solvated PbS-Br (black squares), PbS-EDT (blue triangles), and ZnO (red circles) cQDs in different solvents. The blue shaded area indicates the voltage region where other electrochemical processes occur for PbS cQDs. (D) Plot of *E*_CB_ as a function of the Lewis basicity for PbS-Br. Error bars represent one standard deviation of three independently measured samples. All spectroelectrochemical measurements were performed with LiClO_4_ as the electrolyte under inert atmosphere conditions. The red solid line in (D) is a guide to the eye. The PbS cQDs have a diameter of 5.5 nm with a bandgap of 0.77 eV, and the ZnO cQDs have a bandgap of 3.67 eV.

To determine the influence of the solvent on *E*_CB_, we selected organic aprotic solvents spanning a wide range of relative permittivity (ε) and Lewis basicity (DN, Figure 3A,B). We measured *E*_CB_ for solvated PbS cQD with band gaps of 0.76 eV using the spectroelectrochemical approach described above. As shown in the literature,^33−35^ electrochemical measurements in different solvents require a reference redox system with a reduction potential that does not depend on the nature of the solvent. Changes in the reduction potential with solvents originate from a difference in the Gibbs free energy of solvation between the reduced and oxidated states. Redox couples with a shielded charge are expected to have redox states with a similar Gibbs free energy of solvation and therefore a constant reduction potential in different solvents. It has been previously argued that the Fc/Fc^+^ redox couple is a good candidate because the charge in the central Fe is shielded by the cyclopentadienyl rings (the “ferrocene assumption”).^36^

Quantum chemical calculations of the Fc/Fc^+^ reduction potential in different solvents support this assumption.^33^ To further reinforce this point, we experimentally tested the validity of the “ferrocene assumption” within the solvents investigated here by measuring the Fc/Fc^+^ redox couple reduction potential by cyclic voltammetry with a Ag/AgCl reference electrode in different solvents. The Ag/AgCl reference electrode is isolated from the solvent (this is in contrast to the Ag pseudoreference electrode used during the spectroelectrochemical measurements), therefore preventing shifts in its reduction potential with different solvents, although introducing an unknown liquid junction potential. A change in the measured half-peak potential values with solvent indicates a change of the Fc/Fc^+^ reduction potential or the liquid junction potential. Figure S12 shows that the half-peak potentials of Fc/Fc^+^ are within 100 mV in all of the solvents explored. The only explanation for this negligible effect is either the absence of a solvent effect on both the Fc/Fc^+^ reduction potential and the liquid junction potential or the accidental cancellation of both. Although an accidental cancellation in all six solvents is very unlikely, a measurement of the reduction potential of another redox couple would further reinforce a constant liquid junction in these solvents. Therefore, we also measured the reduction potential of the [Ru(bpy)_3_]^2+/3+^ redox couple in different solvents. This redox couple is also expected to have a reduction potential independent of the solvent because of the large bipyridine rings shielding the central Ru. The reduction potentials of [Ru(bpy)_3_]^2+/3+^ are within 100 mV in all of the measured solvents (Figure S12), further reinforcing that the Fc/Fc^+^ redox couple is a fixed reference system within the explored solvents. Therefore, we selected the Fc/Fc^+^ couple as a fixed reference redox system to be certain that the measured *E*_CB_ values can be compared between different solvents within a fluctuation of about 100 mV.

The obtained values of *E*_CB_ span over a range of nearly 1 eV as shown in Figure 3C. Representative spectroelectrochemical data from which the *E*_CB_ values were extracted is shown in Figures S13–S21. To test how this trend depends on cQD material and surface composition, we determined *E*_CB_ for PbS cQDs with a bandgap of 0.77 eV and capped with ethanedithiol ligands (PbS-EDT, Figure 3C, blue triangles, and Figures S13–S15) and bromide ligands (PbS-Br, Figure 3C, black squares, and Figures S16–S18), as well as for hydroxide and acetate capped ZnO cQDs with a bandgap of 3.67 eV (Figure 3C, red circles, and Figures S19–S21). We also determined E_CB_ in two different electrolyte cations, Li^+^ and tetrabutylammonium (TBA^+^), and found a similar large effect of the solvent with both cations (Figure S22). Moreover, the absolute *E*_CB_ values for both Li^+^ and TBA^+^ are within 100 mV in the same solvent except for acetone. This shows that the shift in *E*_CB_ must come from the interaction between the solvent and the cQD, and not from the solvation of the electrolyte cation.

In line with an earlier report, we find that *E*_CB_ for PbS-Br is ca. 0.3 V more positive than PbS-EDT,^2^ and ca. 0.5 V more positive than ZnO cQDs. While the absolute values of the conduction band energy differ between these samples, the variations of *E*_CB_ with solvent are very similar, which demonstrates the importance of the solvent in determining the conduction band energy. We note that no *E*_CB_ is reported for PbS-EDT in DMSO because it lies outside the stable electrochemical window, stressing the importance of the choice of solvent and ligand for stable electrochemical doping. Moreover, the bandgap was found to be independent of the solvent (Tables S1–S3) and therefore the VB follows the same trend with solvent as the CB.

The large variation of the conduction band energy in different solvents may seem surprising, but it can be understood considering that all of these solvents have some coordinating character to the QD surface. In that sense, solvent molecules are similar to ligands, except that the complexation energy is smaller. Coordination to the surface may induce partial charge transfer between solvent and cQD that may result in shifts of the energy levels, as it is well known for organic molecules,^37^ and has previously been demonstrated for different ligands on PbS cQDs.^2,3^

In line with this reasoning, we found that the *E*_CB_ follows the solvent Lewis basicity, with higher Lewis basicity solvents shifting *E*_CB_ to more negative potentials (Figure 3D). The Lewis basicity is a measure of the tendency of the molecule to donate electron density; as the solvent Lewis basicity increases, the electron density in the cQD increases and the levels move up in energy (i.e., toward more negative potentials). Figure 3D plots the obtained *E*_CB_ for PbS-Br as a function of the Gutmann donor number, an experimental measure of the Lewis basicity.^38^

To test this trend further, we selected two additional solvents with very low (benzonitrile, BN) and very high (*N*,*N*-diethylacetamide, DEA) Lewis basicity (Figure 3B). As expected, these two solvents gave very high and low *E*_CB_ values, respectively (Figure 3D), increasing the range of *E*_CB_ values to nearly 1 eV for the investigated solvents.

### DFT Calculations on cQD Atomistic Models

**Figure 4:**
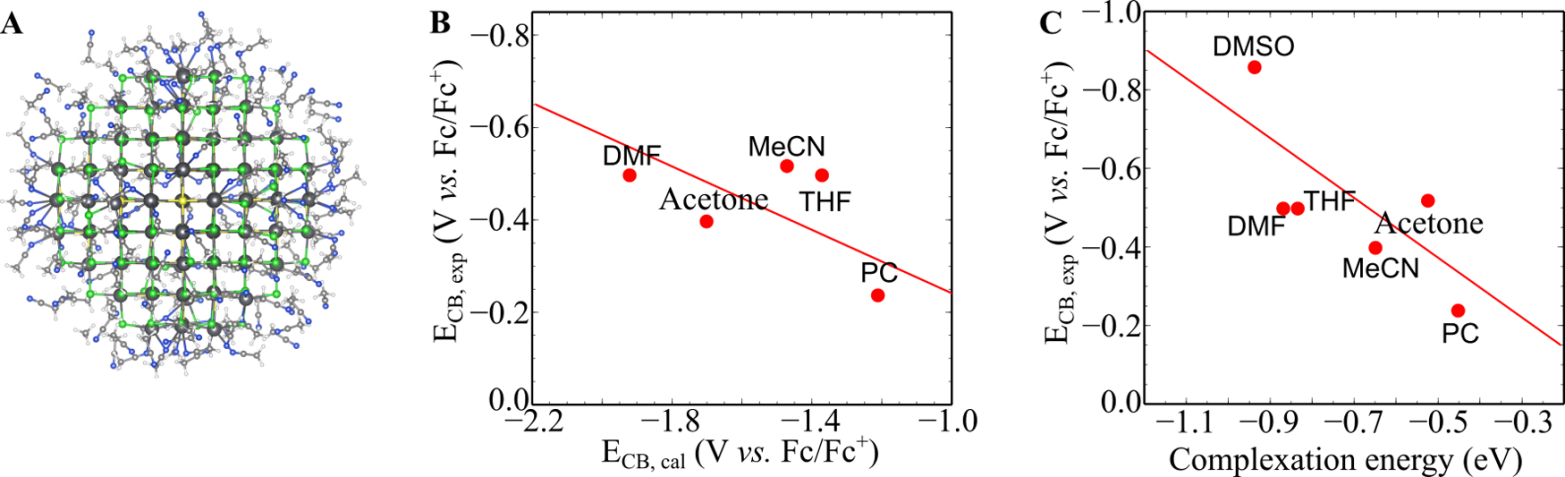
Quantum chemical atomistic model of the band-edge shift by solvation in cQDs. (A) PbS_140_S_85_Cl_110_ is surrounded by the first spherical layer of acetonitrile molecules constructed to simulate the solvated PbS cQD system. (B) Correlation of the calculated conduction band-edge energy (*E*_CB, cal_) in different solvents with the experimental conduction band-edge energy (*E*_CB, exp_). (C) Correlation of *E*_CB, exp_-with the Pb^2+^-solvent complexes’ complexation energy. The red line is a guide to the eye.

To gain further insight into the molecular origin of the experimentally observed *E*_CB_ solvent shift we performed DFT calculations (see the Methods section, Figures S23–26, and Tables S4–S5 for details on the calculations). Our computational model is composed of a PbS cQD with Pb_140_S_85_Cl_110_ stoichiometry corresponding to a diameter of 3.4 nm, and one layer of explicit solvent molecules (Figure 4A). This nonstochiometric model was used to resemble the experimental composition of PbS cQDs,^39^ with excess of Pb atoms passivated by Cl ligands required to keep charge balance and used for computational simplicity,^40^ and is the same as the model used in ref (41), with chloride ligands instead of the iodide ligands used in that work.

We note that using implicit solvent methods did not result in any change of the density of states (DOS) in different solvents, see Figure S24, thus indicating that the trends result from explicit interactions between the solvent molecules and the QDs rather than dielectric effects. Hence, we constructed a 3.45 nm × 3.45 nm × 3.45 nm simulation box of explicit solvent molecules around the cQD and performed molecular dynamics simulations to determine their geometry (see the Methods section for details). After the optimization processes, a smaller model containing only the first solvent layer was extracted. The resulting Pb_140_S_85_Cl_110_ structure with 156 acetonitrile molecules is shown in Figure 4A, and the DOS of this model is shown in Figure S25. To determine *E*_CB_, which is experimentally determined electrochemically, and hence equivalent to the electrochemical potential of electron addition into the CB, we calculated the difference in electronic energy between the neutral and negatively charged cQD, as explained in detail in ref (42).^42^

The predicted *E*_CB_ values of QDs in 5 solvents were found to correlate with the experimental values seen in Figure 4B; the predicted *E*_CB_ in DMSO could not be obtained due to computational convergence issues. We note that the absolute values of *E*_CB_ were not exactly the same as those obtained from the experiment. Such a difference can be attributed to the difference in sizes of the cQDs, the type and number of ligands, and the uncertainty in the DFT calculations. Nonetheless, the theoretical data are qualitatively consistent with our experimentally observed trends in *E*_CB_. Both theoretical and experimental data indicate that solvents have a large influence on the *E*_CB_ of the PbS cQD systems.

We rationalized that the band energy shift originates from specific interactions between surface Pb^2+^ and solvent molecules. In an effort to better understand the nature of the interactions between the solvent molecules and surface Pb^2+^ ions, a series of simple Pb^2+^–solvent complexes were studied (Figure S26). The solvents containing carbonyl groups coordinated through the carbonyl oxygen, while the remainder coordinated through the heteroatom. We next analyzed their interaction with quantum theory of atoms in molecules theory (QTAIM), which quantifies bonding interactions using a topological analysis of the electron density.^43,44^ In all cases, QTAIM analyses revealed bond critical points (BCPs) between Pb^2+^ and the coordinated atom (Figure S26), which are indicative of the presence of a chemical bond. The charge density values ρ_cp_ at the BCPs listed in Table S4 are quite small (∼0.05 au) indicating that interactions between Pb^2+^ and solvent molecules are relatively weak in comparison to a normal C–C bond in acetonitrile (ρ_cp_ = 0.26 au). The interactions are found to be largely noncovalent (as indicated by positive values of the Laplacian of the electron density ∇^2^ρ_cp_) but with some covalent features (as indicated by negative values of the total energy density value *H*_cp_). The relatively small covalent contribution to the total interaction can be quantitatively evaluated through the interaction energy component *E*_covalent_ given in Table S4. The small but significant covalent contribution implies partial charge transfer from the solvent molecules to the Pb^2+^ ion. This partial charge transfer from solvent molecules to Pb^2+^ was also observed from Natural Bond Orbital (NBO) analysis upon formation of Pb^2+^–solvent complexes (Table S5). While these calculations are only for simple model complexes between solvent molecules and Pb^2+^, they support the hypothesis that in the cQD surface coordination of solvent models shifts electron density to the cQD, leading to the observed shift in the energy levels.

Complexation energies calculated from the interaction between Pb^2+^ and individual solvent molecules were found to correlate with the experimental conduction band edges (Figure 4C). Solvents that have stronger interactions with Pb^2+^ tend to result in lower conduction band edges in the cQD systems. This agrees with the trend observed in Figure 4C, as the complexation energy is expected to increase with solvent Lewis basicity. Therefore, the complexation energy between Pb^2+^ and solvent molecules, like the solvent Lewis basicity, provides suitable qualitative descriptors to predict effects of solvents on the conduction band edges of PbS cQDs.

Overall, the experimental and theoretical data lead to the conclusion that the shift of the CB and VB is primarily electrostatic in nature, not covalent, but the magnitude of the shift depends on the strength of the interaction between solvent and surface, which has both covalent and noncovalent contributions. The experimental data show that *E*_CB_ shifts depending on the solvent the cQDs are immersed in (Figure 3C) and that the bandgap remains unchanged (Tables S1–S3). This means that *E*_VB_ shifts in the same direction and by the same quantity as *E*_CB_. As such, this suggests that the effect is electrostatic in nature, as a change in charge density around the cQD would lead to an electrostatic shift that is the same for the conduction and the valence band: an electron in either the CB or VB simply experiences a different electrostatic potential in different solvents, i.e., the inner (or Galvani) potential of the cQD is changed. However, the observed shift does not follow a trend with the dielectric constant and is not observed when DFT/MD calculations are performed with implicit solvents. Instead, the energy level rises with increasing Lewis basicity and is observed when the DFT/MD calculations are performed with explicit solvents. This leads to the conclusion that the shift is due to specific interactions between the solvent and the surface and is not purely dielectric in nature. Based on this information, and our atoms-in-molecules theory analysis of Pb^2+^···solvent coordination bonds (Tables S4 and S5), we hypothesize that the interaction is due to electron donation from the solvent to the cQD, and while largely electrostatic in nature also has a covalent contribution. A strong Lewis base will donate substantial electron density to the 6p orbitals of Pb. The shift in electron density will cause an electrostatic potential change inside the QD for both CB and VB. If an electron in both bands is delocalized similarly over the whole QD, the electrostatic shift will be the same. This is the important effect, and a potential change in the molecular orbital energy of the lowest CB level (1S_e_) due to coupling to the solvent orbitals (i.e., the coupling energy) is much smaller.

## Conclusions

In conclusion, we have measured the band energy levels of cQDs in different solvents by spectroelectrochemistry and found that the band energies of cQDs are critically dependent on the surrounding solvent, resulting in energy shifts of nearly 1 eV for PbS cQDs. This was realized by conceiving stable n-type PbS cQDs. Trends in energy level position are confirmed by DFT calculations, showing that the experimentally observed shifts result from specific interactions between surface metal cation atoms with solvent molecules and scale with the energy of complexation. This complexation results in charge transfer between the solvent and the cQD, resulting in a shift of the energy levels. Therefore, the complexation energy, as well as the solvent Lewis basicity, is found to be a good descriptor to predict the shift in band energy levels of solvated cQDs: the higher the Lewis basicity, the higher the band edges shift in energy. This trend is experimentally observed for cQDs of different materials (ZnO and PbS) and passivated with different ligands (ethanedithiol and bromide), proving to be general behavior. These results are relevant for technologies and fundamental studies that use solvated cQDs or embedded in an environment where coordination to the surface may occur such as a polymer matrix, ranging from solar cells and photocatalysts to light-emitting electrochemical cells and doping engineering.

## Methods

### Materials

All materials were purchased from Sigma-Aldrich unless otherwise stated. Lead(II) oxide (PbO, 99.999%), octadecene (ODE, 90%), bis(trimethylsilyl) sulfide (TMS, synthesis grade), and oleic acid (OA, extra pure, Thermo Fisher Scientific) were used for the synthesis of lead sulfide colloidal quantum dots (PbS cQDs). Zinc acetate (Zn(CH_3_COO)_2_, 99.99%), potassium hydroxide (KOH, 99.99%), ethanol (CH_3_CH_2_OH, dry, max. 0.01% H_2_O), methanol (CH_3_OH, ≥99.8% puriss. p.a.), and hexane (C_6_H_14_, 95% anhydrous) were used for the synthesis of zinc oxide colloidal quantum dots (ZnO cQDs). 1,2-Ethanedithiol (EDT, 98%) and tetrabutylammonium bromide (TBABr, 99%) were used for ligand exchange. Lithium perchlorate (LiClO_4_, 99.99%, dry), tetrabutylammonium perchlorate (TBAClO_4_, 99%), ferrocenium hexafluorophosphate (98%), acetonitrile (MeCN, 99.8% anhydrous), propylene carbonate (PC, 99.7%, anhydrous), dimethylformamide (DMF, 99.8%, anhydrous), dimethyl sulfoxide (DMSO, 99.9%), anhydrous, tetrahydrofuran (THF, 99.9%, anhydrous), benzonitrile (BN, 99%), *N*,*N*-diethylacetamide (DEA, 97%), and acetone (99.8%, anhydrous, VWR) were used for the electrochemical and spectroelectrochemical measurements. Indium tin oxide on glass slides (0.7 mm thick, 7–10 Ohm/Sq) was purchased from MSE Supplies and used as substrate for the cQD films.

### cQD Synthesis, Film Assembly, and Ligand Exchange

ZnO cQDs were synthesized following a previously described procedure.^9^ Zinc acetate (0.628 g) was dissolved in ethanol (50 mL) by heating the solution to 60 °C while stirring. When dissolved, a solution of KOH (0.351 g) in methanol (5 mL) was added dropwise (ca. 1 drop per second), and the solution was taken out of the heat. The ZnO cQDs were isolated from the reaction mixture by adding hexane until the solution became turbid and centrifuged, the hexane removed, and the cQDs redispersed in 6 mL of ethanol. The cQD dispersion was stored at −20 °C. ZnO cQD films were formed by drop-casting the cQD dispersion (50 μL) onto indium tin oxide on glass slides (1 × 2.3 cm^2^) and annealed at 60 °C for 1 h.

PbS cQDs were synthesized following a previously described procedure.^45^ In a typical synthesis, lead(II) oxide (90 mg) was dissolved in OA (0.25 mL) and ODE (3 mL) by heating under vacuum to 100 °C for 1 h. The temperature was then set to the desired temperature (e.g., 150 °C), and a solution of TMS (42 μL) in ODE (0.75 mL) was injected under a nitrogen atmosphere. The heating mantle was lowered away from direct contact with the reaction flask immediately after injection of the TMS solution and allowed to cool to room temperature. The PbS cQD size was adjusted by modifying the OA/Pb/S ratio (from 4:2:1 to 80:2:1) and temperature (115–150 °C). The PbS cQDs were isolated from the reaction mixture by adding acetone until the solution became turbid, centrifuged, the supernatant removed, and the cQDs redispersed in 8 mL of hexane. The cQD dispersion was stored at room temperature. PbS cQD films were formed by drop-casting the cQD dispersion (25 μL) onto indium tin oxide on glass slides (1 × 2.3 cm^2^) and let dry to room temperature. The dry films were immersed into the ligand exchange solution (EDT 0.1 M in MeCN or TBABr 0.1 M in MeOH) for 1 min and thoroughly rinsed with MeCN or MeOH, respectively. The cQD storage, film assembly, and ligand exchange were performed under an inert atmosphere (water <0.5 ppm and oxygen <0.1 ppm).

### Spectroelectrochemical Measurements

Spectroelectrochemical measurements were performed with an Autolab PGSTAT128N potentiostat in a three-electrode electrochemical cell setup with a platinum sheet as the counter electrode, a silver wire as the pseudoreference electrode, and the cQD films as the working electrodes. The absorbance changes were measured as a function of the applied electrochemical potential with a fiber-based UV–vis spectrometer (Ocean Optics USB2000) using an Ocean Optics DH 2000 lamp as a light source. A solution of 0.1 mol L^–1^ LiClO_4_ (unless otherwise stated) in the specified solvent was deoxygenated by purging argon gas (99.999%) for >20 min and used as electrolyte. The pseudoreference electrode was calibrated throughout the course of the experiments against the ferrocene/ferrocenium (Fc/Fc^+^) redox couple in each solvent (Figure S27) using a polycrystalline gold working electrode. The gold electrode was used because of the more ideal behavior of Fc/Fc^+^ on this electrode. All procedures were performed inside a glovebox with a water content <0.5 ppm and an oxygen content <0.1 ppm.

### Characterization Methods

Transmission electron microscopy images were acquired using a JEOL JEM1400 transmission electron microscope operating at 120 keV. Steady-state absorption spectra of PbS cQDs were recorded using a PerkinElmer Lambda 1050 UV/vis/NIR spectrophotometer. The XPS measurements were performed under UHV (<2 × 10^–7^ mbar) on a Thermo Fisher K-Alpha equipped with an Al Kα source.

### Computational Methods

Density functional theory (DFT) calculations were used to predict the density of states (DOSs) and associated band gaps of a series of model quantum dots (Figure S23) in acetone, acetonitrile (MeCN), dimethylformamide (DMF), dimethyl sulfoxide (DMSO), tetrahydrofuran (THF), and propylene carbonate (PC), using a variety of methods to simulate the solvent environment. Initially, a simple implicit solvent model was used to model the effects of solvents on the density of states of a simple Pb_80_S_80_ quantum dot (see Figure S23a). Geometries were optimized at the PBE^46,47^/def2svp^48^ level of theory using Gaussian 16,^49^ and the SMD^50^ method was used to simulate the environment; corresponding gas phase calculations were also performed for comparison. However, using this approach, the predicted densities of states were found to be independent of the solvent environment, in contradiction with experiment and with our calculations using explicit solvents (Figure S24). As noted above, this indicates that implicit solvent models cannot be used to study the effects of solvents on electronic structures of PbS quantum dots, and explicit solvents were thus used for the remainder of this work.

Next, a larger, more realistic QD model including Cl^–^ counterions, Pb_140_S_85_Cl_110_, was studied in a simulation box (3.45 nm × 3.45 nm × 3.45 nm) containing explicit solvent molecules (260 acetone, 345 MeCN, 219 DMF, 242 DMSO, 242 PC, and 204 THF molecules, Figure S23b for visualization of MeCN). Molecular dynamics (MD) simulations (1 ns of NPT, 1 ns of annealing, and 3 ns of NVT) were first run to allow the solvent molecules to relax and to avoid nonphysical geometries, while the PbS QDs were kept frozen. The OPLS-AA force field^51^ obtained from the LigParGen server^52^ was used for all solvent molecules during the MD simulations, which were performed in Gromacs.^53^ All details of force field parameters and MD simulation parameters can be obtained from the Supporting Information. After an equilibrium NVT simulation of 3 ns, the last frame was extracted from the MD trajectory and used as the initial coordinate in the subsequent quantum chemical optimizations. The full simulation box was first geometrically optimized with the Quickstep DFT module implemented in the CP2K program.^54^ The PBE functional^46,47^ with DFTD3(BJ)^54^ dispersion correction scheme was employed in combination with the DZVP-MOLOPT-SR-GTH basis set^53^ and GTH-PBE pseudopotential.^55,56^ Due to large system sizes (ca. 2500 atoms), a plane-wave cutoff was set to 400 Ry in conjunction with a Gaussian mapping cutoff of 55 Ry to make the DFT optimization feasible and as accurate as possible. The cell dimensions were also optimized with respect to an external reference pressure of 1 atm. After the optimization processes, a smaller model of QDs containing only the first solvent layer (Figure S23c) was extracted from the geometrically relaxed QD systems obtained from the previous DFT optimization processes and one electron was injected for conduction band-edge energy calculations.

Model complexes formed between Pb^2+^ and single solvent molecules were also probed theoretically to study the nature of the interaction between the solvents and PbS QDs. Electronic interaction energies between Pb^2+^ and solvent molecules were evaluated at wB97XD/ma-def2tzvp//wB97XD/def2sv(p),^48,57^ and the quantum theory of atoms in molecules (QTAIM)^43,44^ analysis implemented in ADF 2022^58^ was conducted to better understand the interaction between Pb^2+^ and solvent molecules. The QTAIM calculations were used to obtain the density (ρ_cp_) and Laplacian of the density (∇^2^ρ_cp_) at bond critical points between the metals in the studied complexes. These in turn were used to obtain the total energy density H_cp_ associated with the corresponding bonding interaction via eq 2.
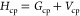
2where *G*_cp_ is the kinetic energy density at a BCP in the approximation of Abramov^59^ and *V*_cp_ is the potential energy density at the same BCP. *G*_cp_ and *V*_cp_ were calculated using eqs 3 and 4, respectively.^60^
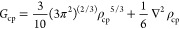
3
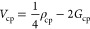
4

Covalent and noncovalent energies of the interaction between Pb^2+^ and solvent molecules were calculated using the interacting quantum atoms (IQA) method^61^ with the aid of the ADF program. Natural bond orbital (NBO)^62^ analysis was conducted to extract charges on the Pb atom in the Pb^2+^-solvent complexes so as to assess the amount of charge transfer between Pb and the solvent. The complexation energy was calculated from the pure electronic energies of fragments using the counterpoise procedure^63^ implemented in Gaussian 16. The SMD (for calculations using Gaussian 16) and COSMO (for calculations using ADF) methods were used to treat implicit solvent effects in all cases.

## Supporting Information Available

Origins of the spectroelectrochemical features; stability of PbS cQDs; electron distribution in energy in cQDs; size-dependent properties of PbS cQDs; reference redox system; solvent-dependent properties of PbS-EDT, PbS-Br, and ZnO cQDs; computational calculations; pseudoreference electrode calibration; and bond critical point data (PDF)

## Abbreviations

cQD: colloidal quantum dot
CB: conduction band
VB: valence band
WE: working electrode
CE: counter electrode
RE: reference electrode
OCP: open-circuit potential
*E*_CB_: conduction band edge
*E*_VB_: valence band edge
Fc: ferrocene
Fc^+^: ferrocenium
*E*_F_: Fermi level
*E*_PbS/LixPbS_: lithiation potential
*E*_PbS/S_: oxidative degradation potential
fwhm: full width at half-maximum
MeCN: acetonitrile
DMSO: dimethyl sulfoxide
DMF: dimethylformamide
THF: tetrahydrofuran
PC: propylene carbonate
BN: benzonitrile
DEA: *N*,*N*-diethylacetamide
BCP: bond critical point
EDT: ethanedithiol
