# Supplementary material for: Solvation Shifts the Band-Edge Position of Colloidal Quantum Dots by Nearly 1 eV
Source: J Am Chem Soc. 2024 Mar 26;146(14):9928–38. doi: 10.1021/jacs.4c00402 (PMC11009959; doi:10.1021/jacs.4c00402)
Supplement: Supplementary file 1 — ja4c00402_si_001.pdf [file ja4c00402_si_001.pdf]

# Supporting Information for

# Solvation shifts the band-edge position of colloidal quantum dots by nearly 1 eV

Yan B. Vogel,[*,†], Le Nhan Pham,[‡] Maarten Stam[†], Reinout F Ubbink[†], Michelle L. Coote[‡], Arjan J. Houtepen[†]

[†]*Department of Chemical Engineering, Delft University of Technology, Van der Maasweg 9, 2629 HZ Delft, The Netherlands*

[‡]*Institute for Nanoscale Science and Technology, College of Science and Engineering, Flinders University, Bedford Park, South Australia 5042, Australia*

## Table of Contents: **Pg.**

***Figure S1. Origins of the spectroelectrochemical features. ----------------------------- 2***

***Figures S2-S6. Stability of PbS cQDs. ------------------------------------------------------ 3-5***

***Figure S7. Electron distribution in energy in cQDs. -------------------------------------- 6***

***Figures S8-S11. Size dependent properties of PbS cQDs. -------------------------------- 7-9***

***Figure S12. Reference redox system. ------------------------------------------------------ 9***

***Figures S13-S15 and Table S1. Solvent dependent properties of PbS-EDT cQDs.-- 10-12***

***Figures S16-S18 and Table S2. Solvent dependent properties of PbS-Br cQDs.----- 13-15***

***Figures S19-S21 and Table S3. Solvent dependent properties of ZnO cQDs. -------- 16-18***

***Figure S22. Effect of the cation in the conduction band energy level. ---------------- 19***

***Figure S23-S26 and Tables S4-S5. Computational calculations. ---------------------- 20-22***

***Figures S27. Pseudoreference electrode calibration. ------------------------------------ 23***

***Appendix. Bond critical point (BCP) data. --------------------------------------------- 24-30***

# Origins of the spectroelectrochemical features

Figure S1A shows the ΔA spectrum of PbS cQDs at -0.85 V and -1.05 V vs. Fc/Fc+. This data is retrieved from the "spectral slices" of Figure 1H on the main text. When the potential is scanned negative, first a single bleach appears centered at 1630 nm. The same feature appears when the potential is scanned positive, as shown in Figure S1C (data retrieved from the "spectral slices" of Figure 1F on the main text). In both cases, this bleach corresponds to the $1S_h1S_e$, as the $1S_e$ level (or $1S_h$ level for positive potentials) is populated with electrons (holes). The absence of a second bleach demonstrates the absence of the $1S_h1P_e$ transition. As the potential is scanned more negative, the bleach at 1630 nm saturates, demonstrating complete filling of the $1S_e$. level (Figure S1B). As the 1630 nm bleach saturates, two additional bleaches appear centered at 1220 nm and 1400 nm. The bleach at 1220 nm corresponds to the $1P_h1P_e$ transition, as the $1P_e$ level is populated with electrons. The 1400 nm bleach follows the same potential dependence as the 1220 nm bleach, indicating that also arises because of population of the $1P_e$ level. This can only mean that the 1400 nm bleach corresponds to the $1S_h1P_e$ transition.

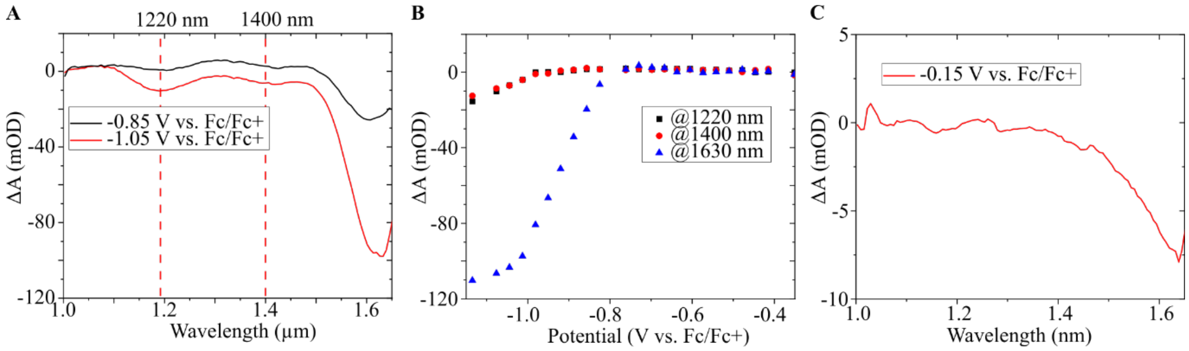


**Figure S1. A)** ΔA spectrum at -0.85 V vs Fc/Fc+ (black solid line) and -1.05 V vs Fc/Fc+ (red solid line), **B)** ΔA vs. voltage at 1220 nm (black squares), 1400 nm (red circles), and 1630 nm (blue triangles), and **C)** ΔA spectrum at -0.15 V vs Fc/Fc+ of PbS cQDs.

## Stability of PbS cQDs

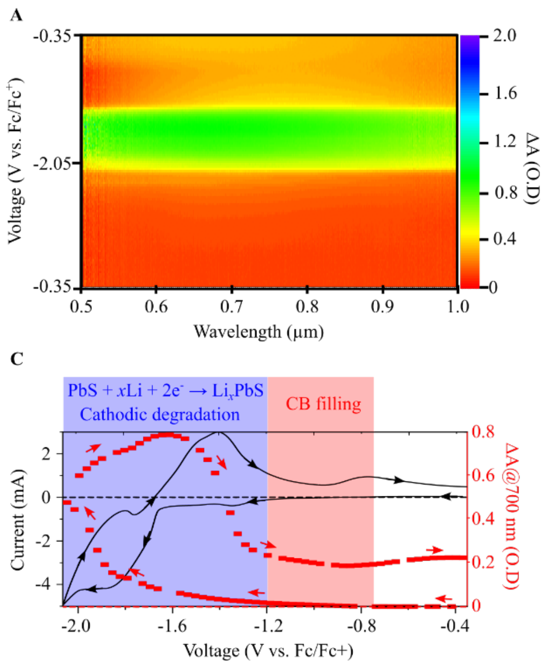


**Figure S2.** Voltage–wavelength–ΔA of **A)** PbS-EDT cQDs with a bandgap of 0.78 eV and **B)** blank ITO electrode. **C)** Cyclic voltammograms (black solid and dashed line) and ΔA vs. voltage at 700 nm (red symbols and red dashed line) of PbS-EDT cQDs with a bandgap of 0.78 eV and blank ITO, respectively. The measurements were performed in acetonitrile with 0.1 M $LiClO_4$ as supporting electrolyte.

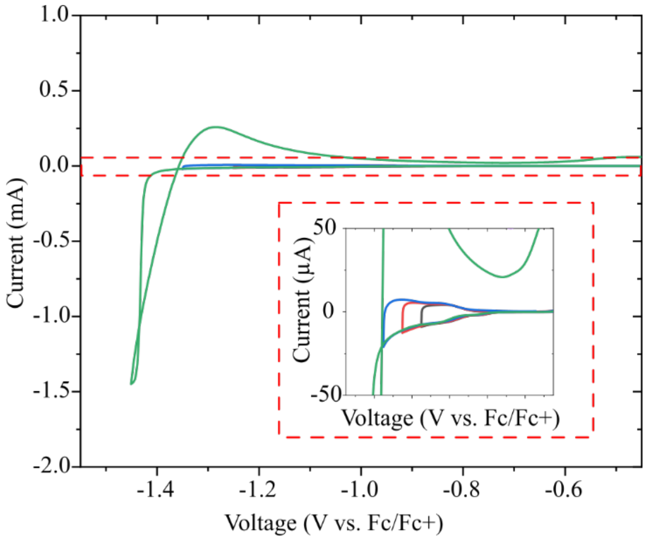


**Figure S3.** Cyclic voltammetry (20 mV/s) of PbS-EDT at increasingly negative potential. The measurements were performed in acetonitrile with 0.1 M $LiClO_4$ as supporting electrolyte.

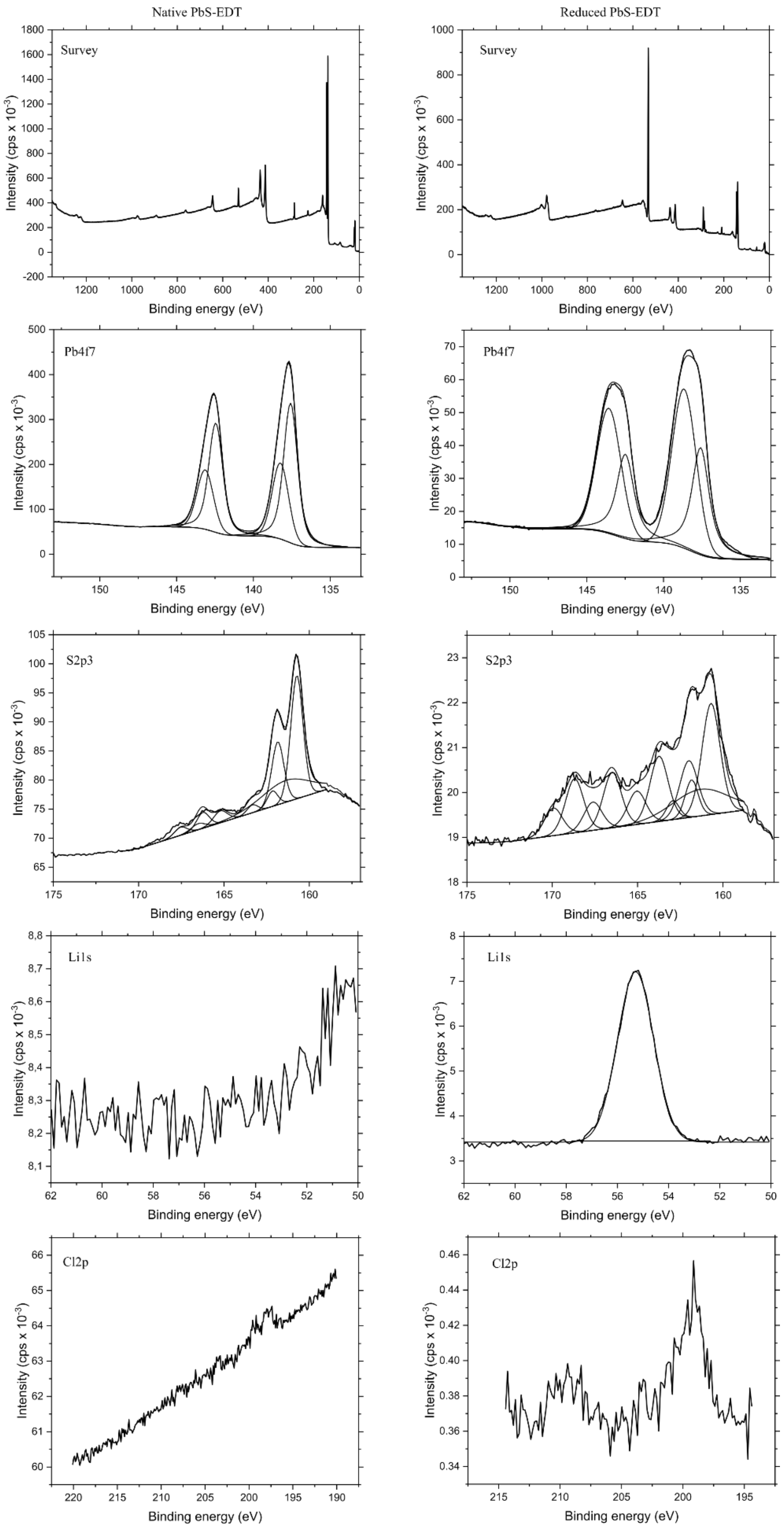


**Figure S4.** X-ray photoelectron spectroscopy of as-prepared (left) and reduced (right) PbS-EDT cQDs. The samples were reduced by applying a constant voltage of -1.95 V vs. Fc/Fc+ for 1 min. The as-prepared samples were immersed in the electrolyte. In both cases

the samples were thoroughly rinsed in acetonitrile and dried under vacuum before the XPS measurements. The atomic composition ratio of Pb:S:Li changes from 1:1:0 for the native PbS-EDT to 1:1:30 for the reduced PbS-EDT sample.

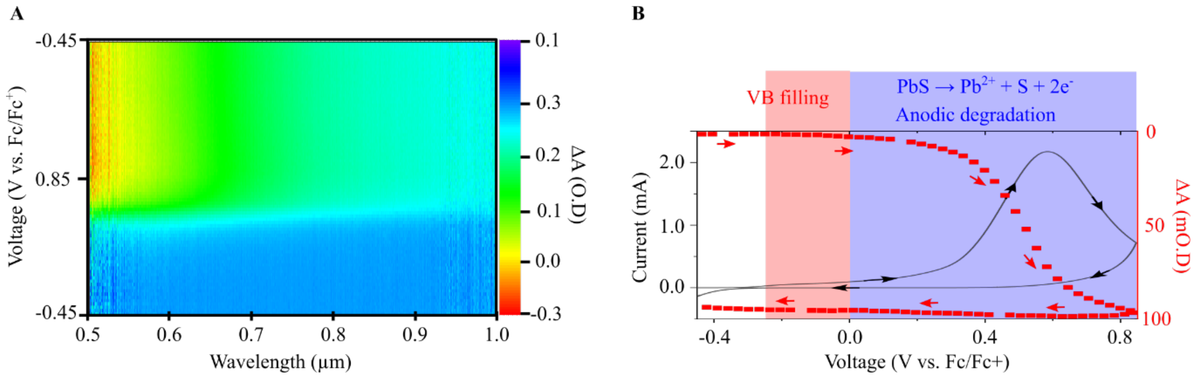


**Figure S5. A)** Voltage–wavelength–ΔA and **B)** ΔA (at 700 nm) vs. voltage plots of PbS-EDT cQDs with a bandgap of 0.78 eV. The measurements were performed in acetonitrile with 0.1 M $LiClO_4$ as supporting electrolyte.

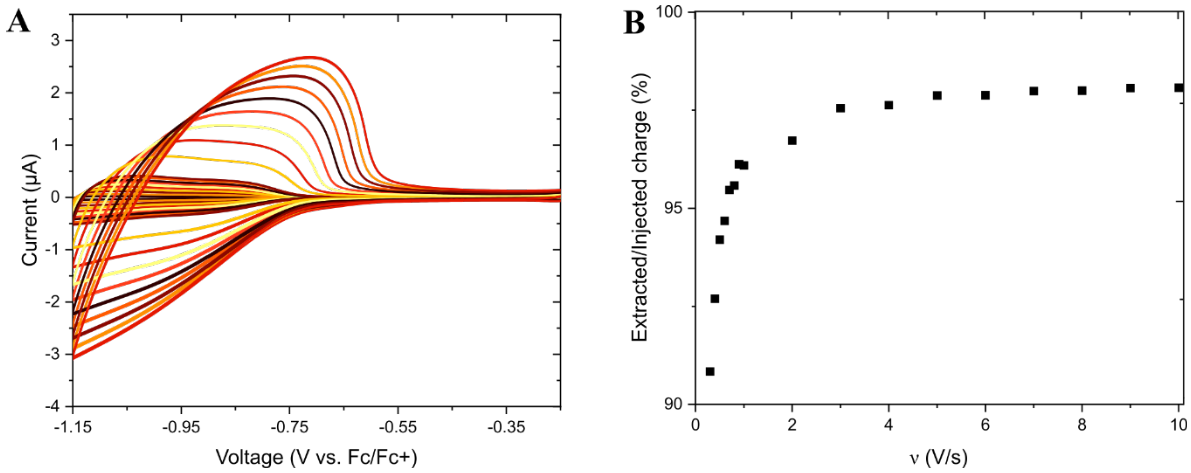


**Figure S6. A)** Cyclic voltammetry at different scan rates (0.1 to 10 V/s) of PbS-EDT. **B)** Ratio of the integrated anodic/cathodic current as a function of the scan rate. The measurements were performed in acetonitrile with 0.1 M $LiClO_4$ as supporting electrolyte.

## Electron distribution in energy in cQDs

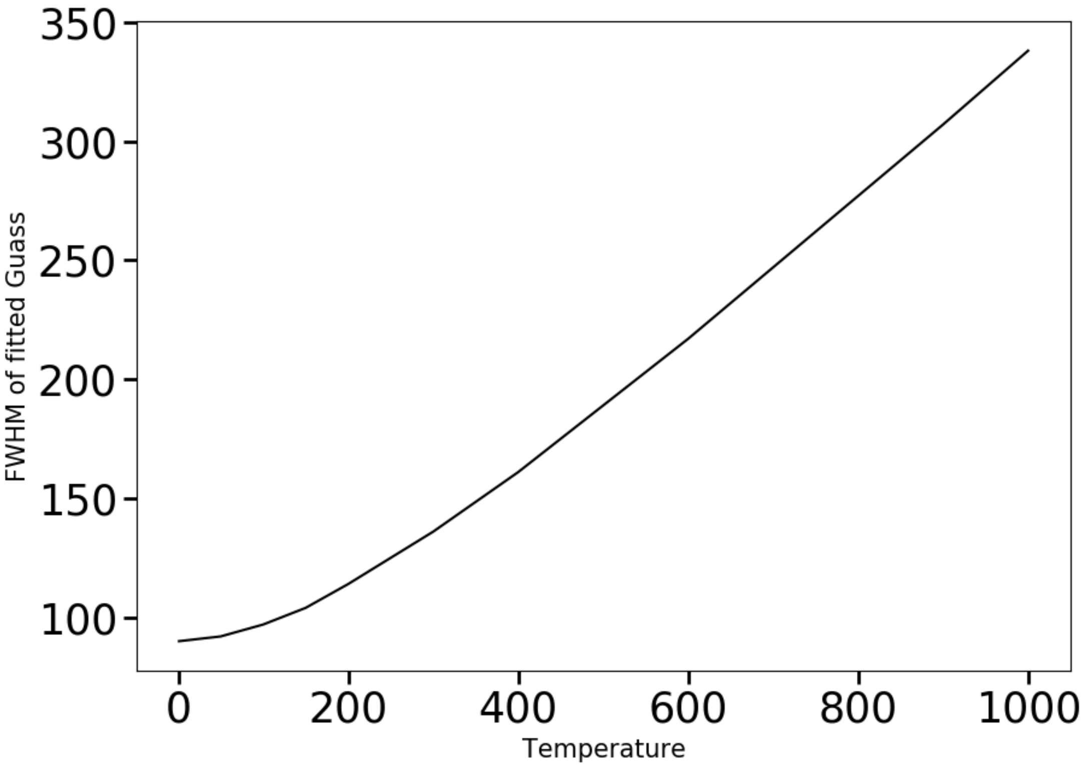


**Figure S7.** Full width half maximum of the fitted Gaussian of the electron distribution as a function of the temperature. The electron distribution was calculated from equation (1) in the main text.

# Size dependent properties of PbS cQDs

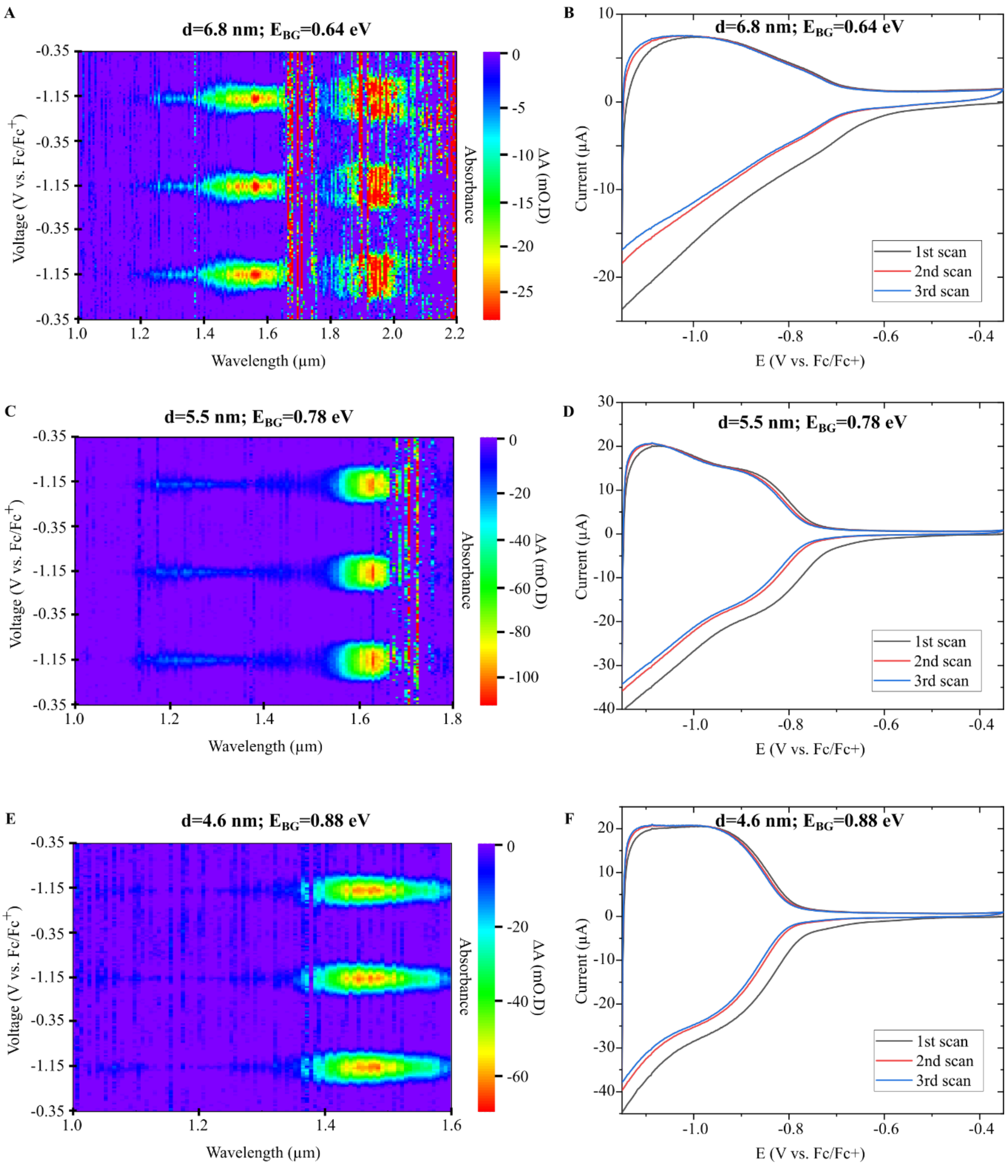


**Figure S8. A), C), E)** Voltage–wavelength–ΔA and **B), D), F)** cyclic voltammetry (50 mV/s) plots of PbS-EDT cQDs with different bandgaps by varying the cQD size. The measurements were performed in acetonitrile with 0.1 M $LiClO_4$ as supporting electrolyte. The cQD diameter (d) and bandgap ($E_{BG}$) are specified above each plot.

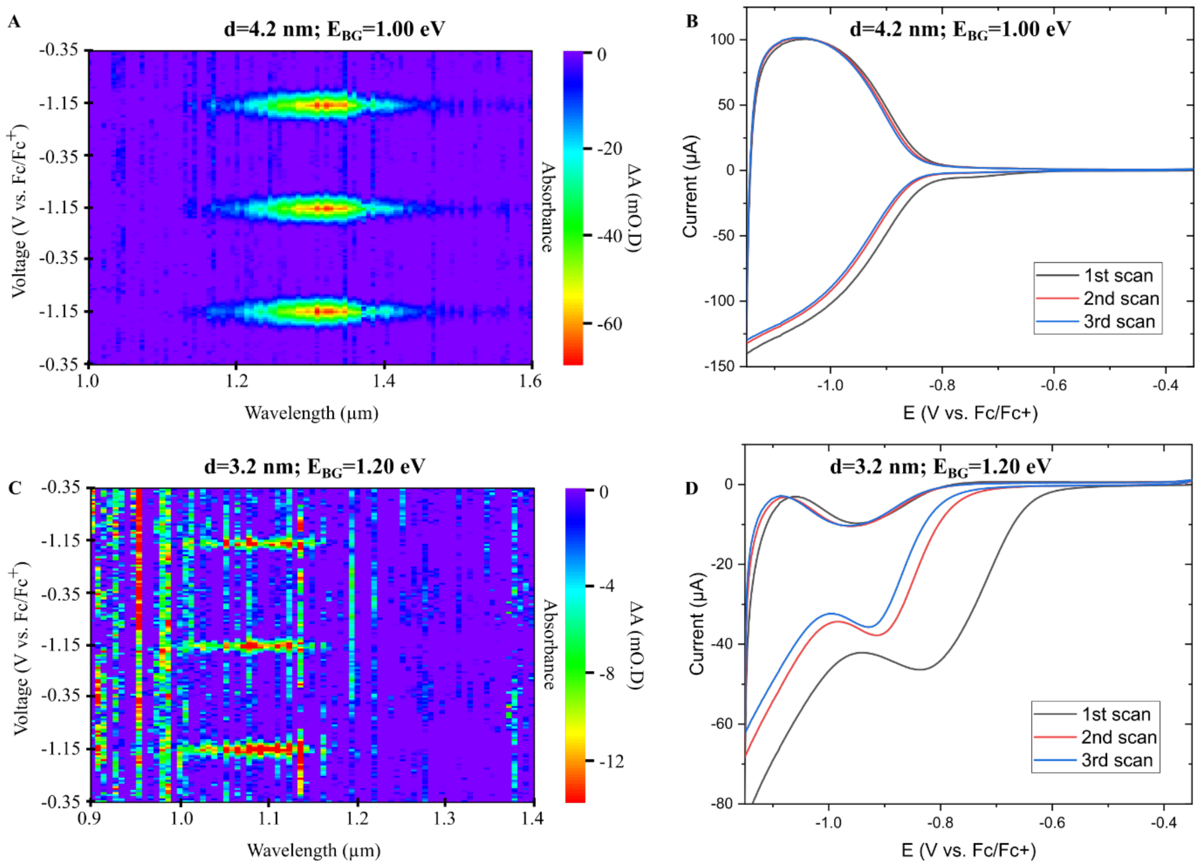


**Figure S9. A), C)** Voltage–wavelength–ΔA and **B), D)** cyclic voltammetry (50 mV/s) plots of PbS-EDT cQDs with different bandgaps by varying the cQD size. The measurements were performed in acetonitrile with 0.1 M $LiClO_4$ as supporting electrolyte. The cQD diameter (d) and bandgap ($E_{BG}$) are specified above each plot.

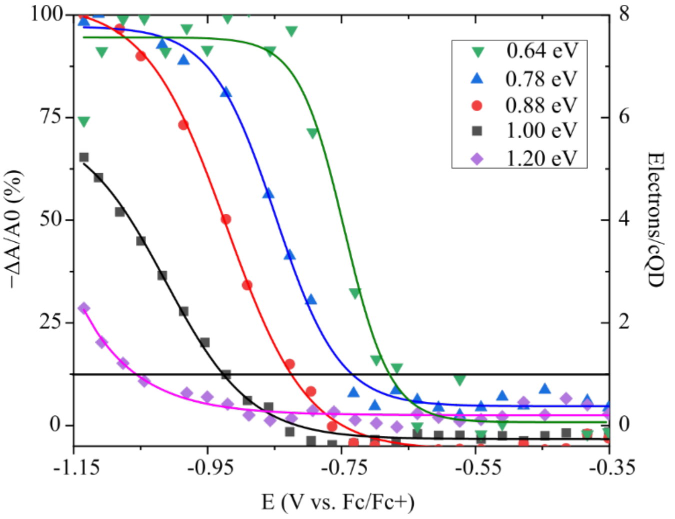


**Figure S10.** $\Delta A/A_0$–voltage plot of the $1S_h$-$1S_e$ transition for PbS-EDT cQDs with different bandgaps (see inset) by varying the cQD size. The measurements were performed in acetonitrile with 0.1 M $LiClO_4$ as supporting electrolyte. Lines are fits to a Boltzmann distribution function.

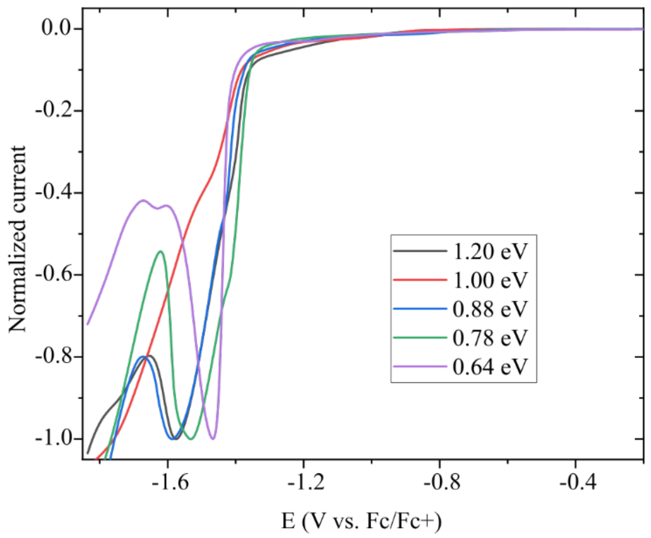


**Figure S11.** Linear sweep voltammograms (normalized to the peak current) of PbS-EDT with different bandgaps (see inset) by varying the cQD size. The strong increase in current around -1.4V is attributed to lithiation, which appears to be size-independent. The measurements were performed in acetonitrile with 0.1 M $LiClO_4$ as supporting electrolyte.

# Reference redox system.

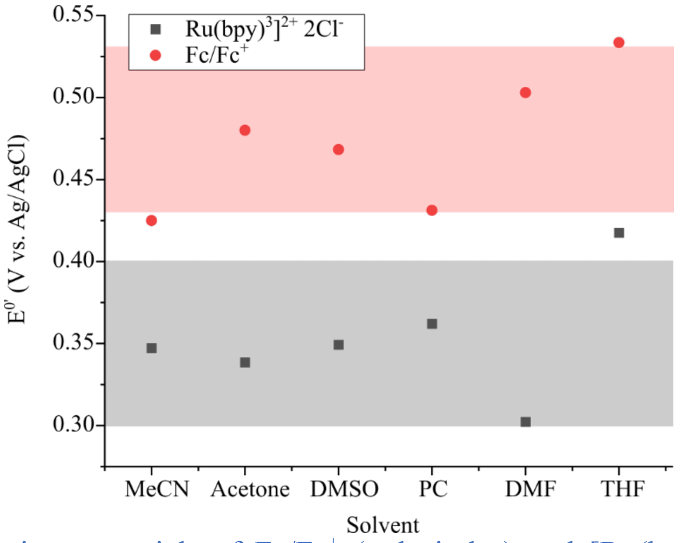


**Figure S12.** Reduction potentials of $Fc/Fc^{+}$ (red circles) and $[Ru(bpy)_3]^{2+}\ 2Cl^{-}$ (black squares) for the solvents explored in this work, as measured by cyclic voltammetry with a Ag/AgCl reference electrode. All values are within ~100 mV as indicated by the colored boxes, strongly suggesting a small effect of the solvent on the liquid junction potential and reduction potential of the redox couples.

# Solvent dependent properties of PbS-EDT cQDs

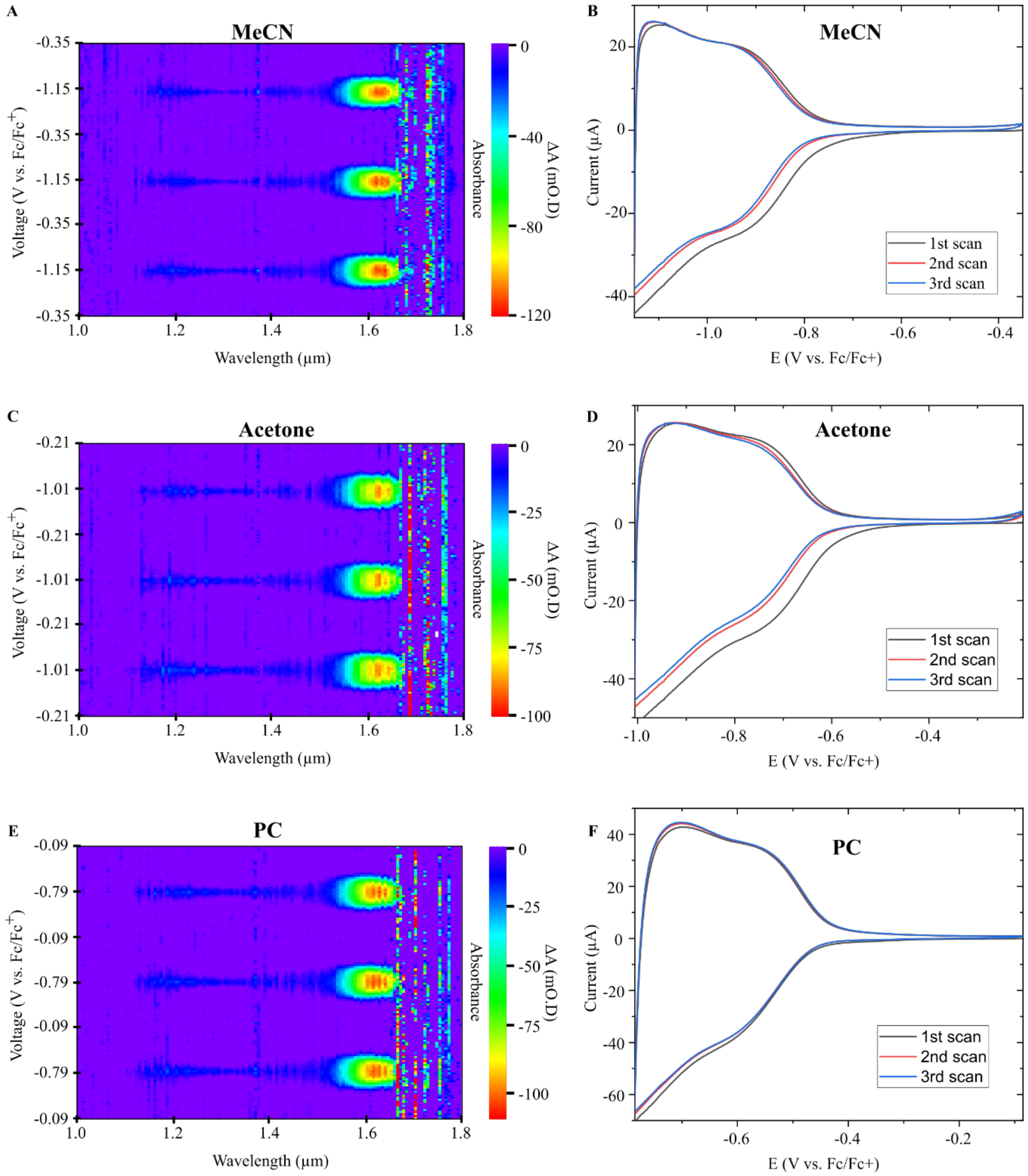


**Figure S13. A)**, **C)**, **E)** Voltage–wavelength–ΔA and **B)**, **D)**, **F)** cyclic voltammetry (50 mV/s) plots of PbS-EDT cQDs with a bandgap of 0.77 eV solvated with different solvents. The solvent used is indicated above each plot. The measurements were performed with 0.1 M $LiClO_4$ as supporting electrolyte. The solvent used is specified above each plot.

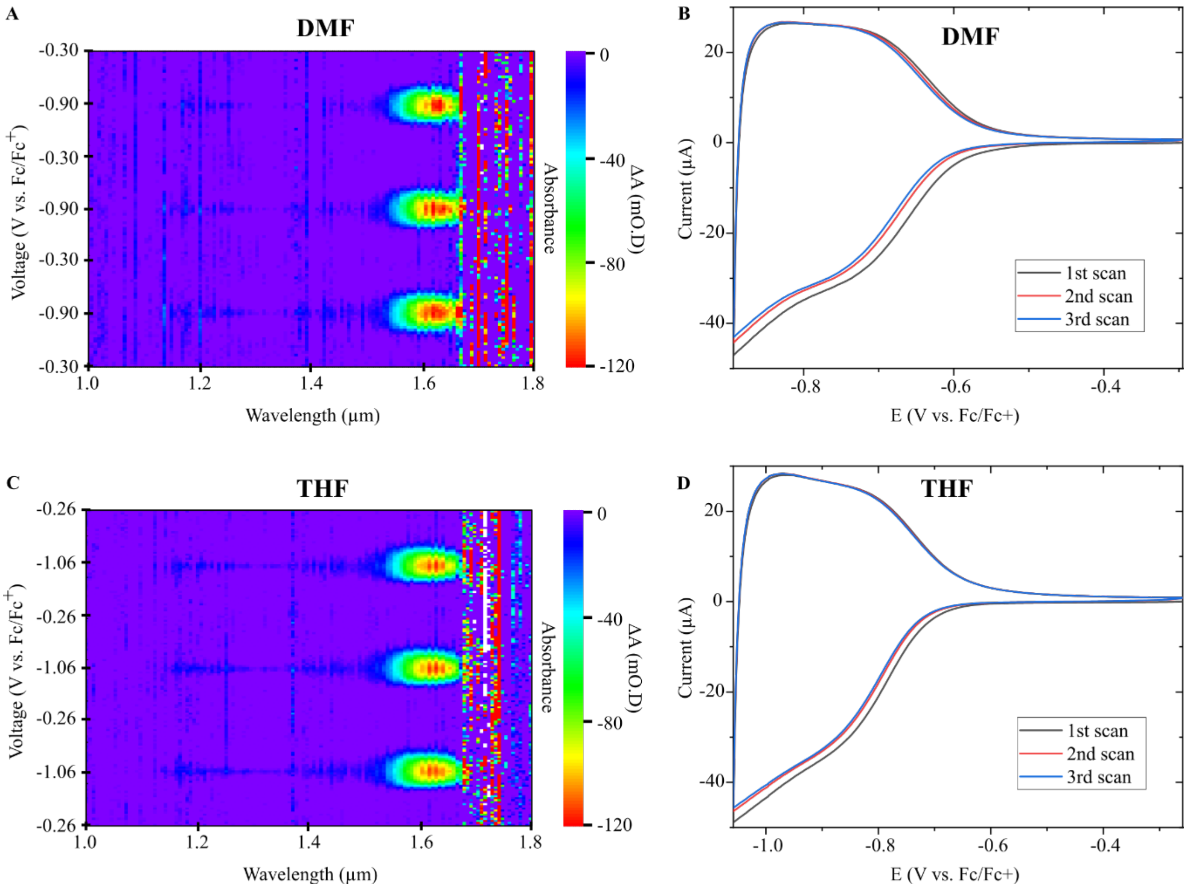


**Figure S14. A), C)** Voltage–wavelength–ΔA and **B), D)** cyclic voltammetry (50 mV/s) plots of PbS-EDT cQDs with a bandgap of 0.77 eV solvated with different solvents. The solvent used is indicated above each plot. The measurements were performed with 0.1 M $LiClO_4$ as supporting electrolyte. The solvent used is specified above each plot.

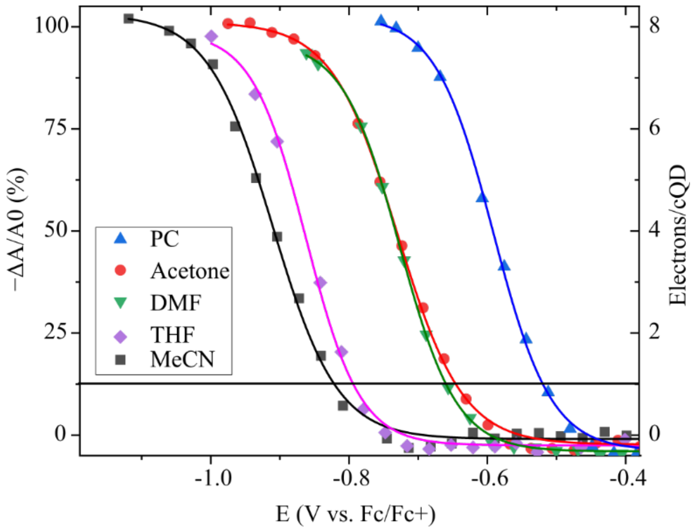


**Figure S15.** $\Delta A/A_0$–voltage plot of the $1S_h$-$1S_e$ transition for PbS-EDT cQDs with a bandgap of 0.77 eV solvated with different solvents. The measurements were performed with 0.1 M $LiClO_4$ as supporting electrolyte. Lines are fits to a Boltzmann distribution function.

| Solvent | Bandgap (eV) |
|---|---|
| THF | 0.77 |
| PC | 0.77 |
| MeCN | 0.77 |
| DMF | 0.77 |
| Acetone | 0.77 |

**Table S1. PbS-EDT bandgap in different solvents.** The bandgap was measured from the position of the $1S_h$-$1S_e$ transition peak from the differential absorbance spectra for PbS-EDT films immersed in different solvents.

## Solvent dependent properties of PbS-Br cQDs

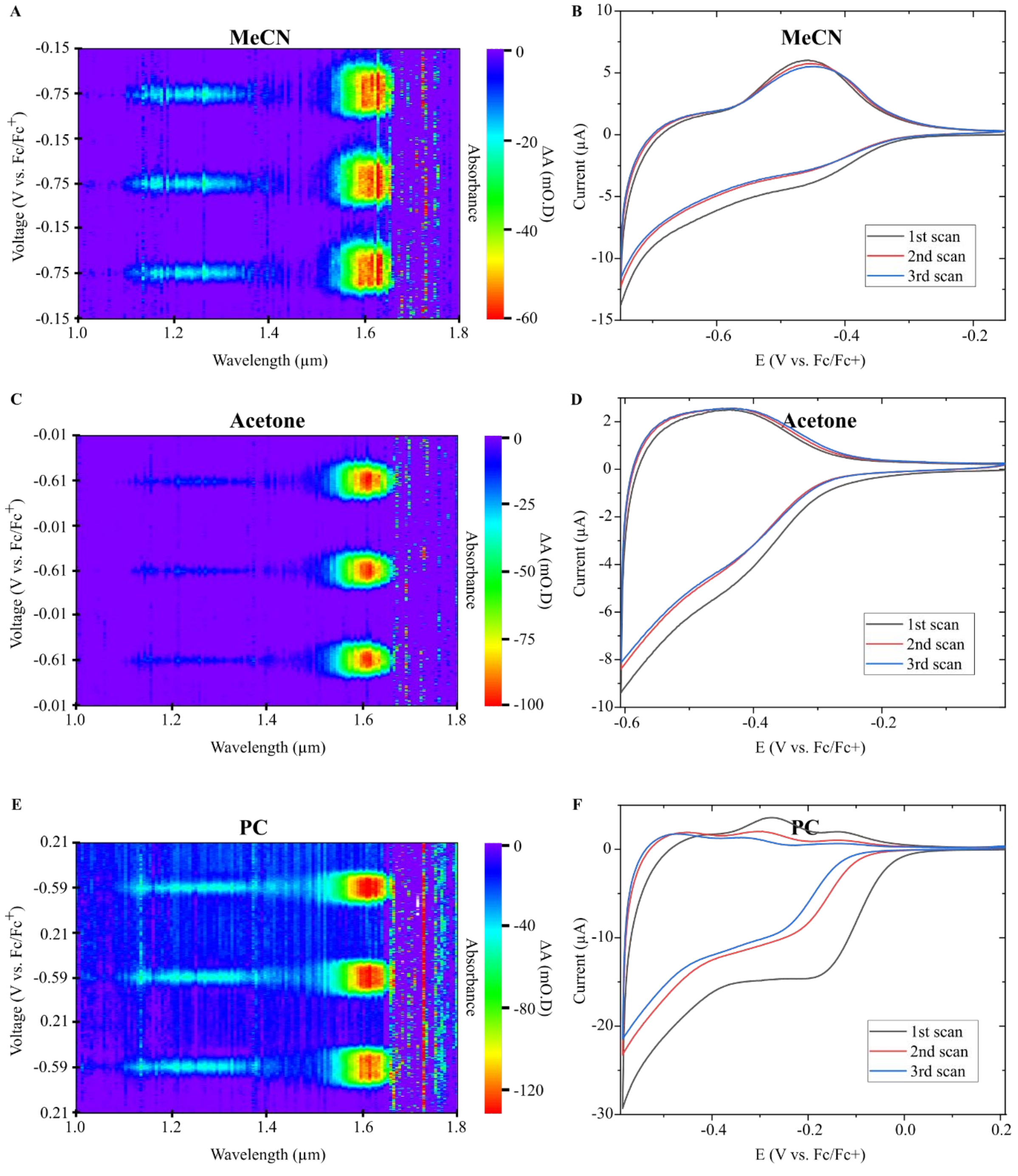


**Figure S16. A), C), E)** Voltage–wavelength–ΔA and **B), D), F)** cyclic voltammetry (10 mV/s) plots of PbS-Br cQDs with a bandgap of 0.77 eV solvated with different solvents. The solvent used is indicated above each plot. The measurements were performed with 0.1 M $LiClO_4$ as supporting electrolyte.

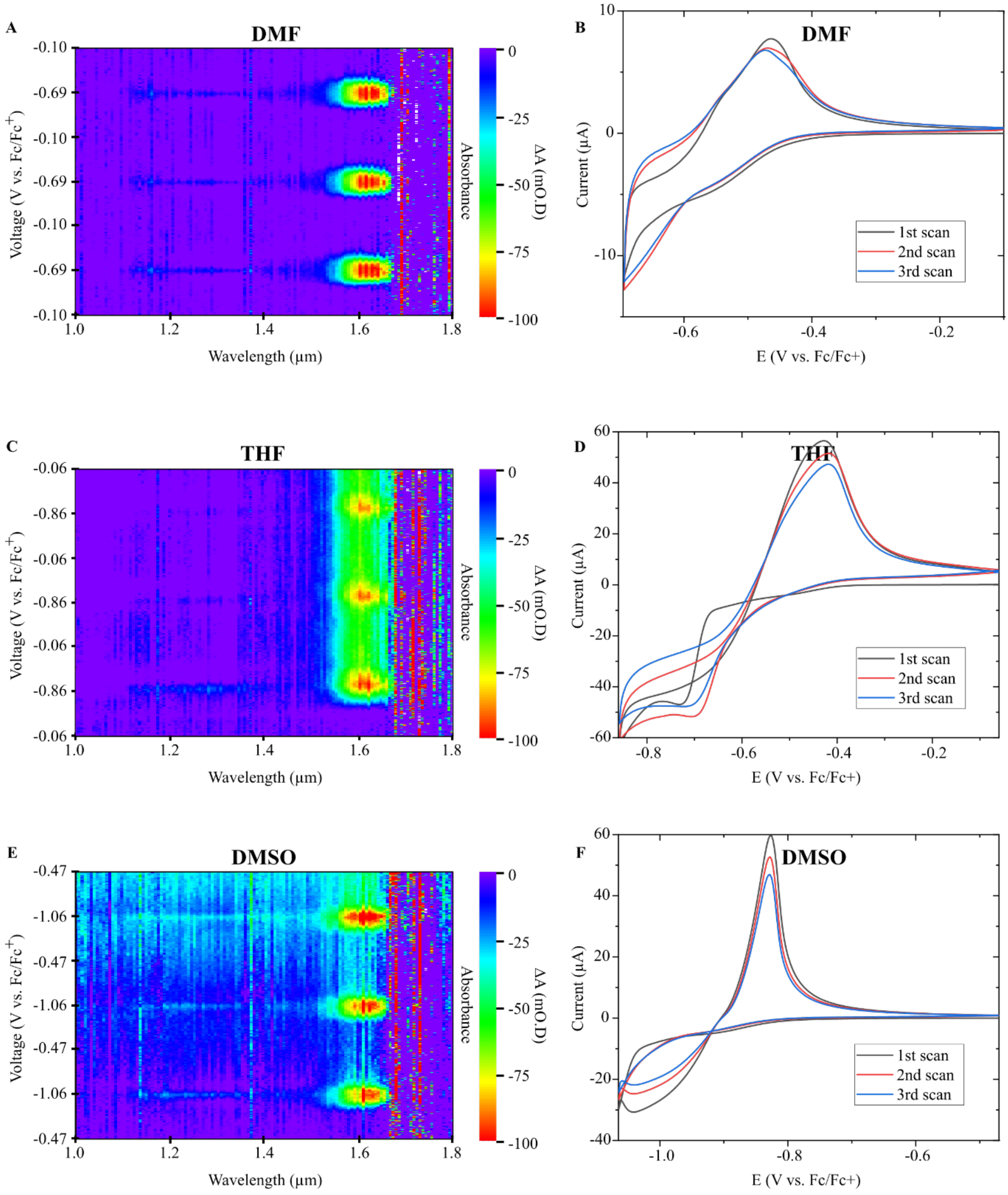


**Figure S17. A)**, **C)**, **E)** Voltage–wavelength–ΔA and **B)**, **D)**, **F)** cyclic voltammetry (10 mV/s) plots of PbS-Br cQDs with a bandgap of 0.77 eV solvated with different solvents. The solvent used is indicated above each plot. The measurements were performed with 0.1 M $LiClO_4$ as supporting electrolyte.

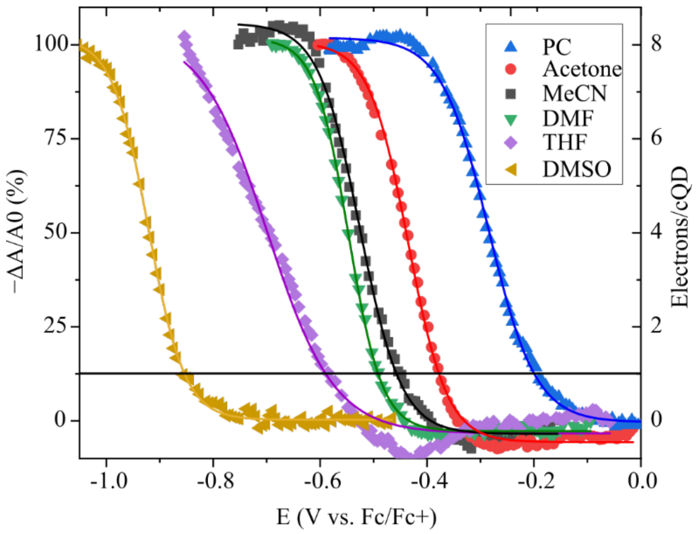


**Figure S18.** $\Delta A/A_0$–voltage plot of the $1S_h$-$1S_e$ transition for PbS-Br cQDs with a bandgap of 0.77 eV solvated with different solvents. The measurements were performed with 0.1 M $LiClO_4$ as supporting electrolyte. Lines are fits to a Boltzmann distribution function.

| **Solvent** | **Bandgap (eV)** |
|---|---|
| THF | 0.77 |
| PC | 0.77 |
| MeCN | 0.77 |
| DMSO | 0.77 |
| DMF | 0.77 |
| Acetone | 0.77 |

**Table S2. PbS-Br bandgap in different solvents.** The bandgap was measured from the position of the $1S_h$-$1S_e$ transition peak from the differential absorbance spectra for PbS-Br films immersed in different solvents.

## Solvent dependent properties of ZnO cQDs

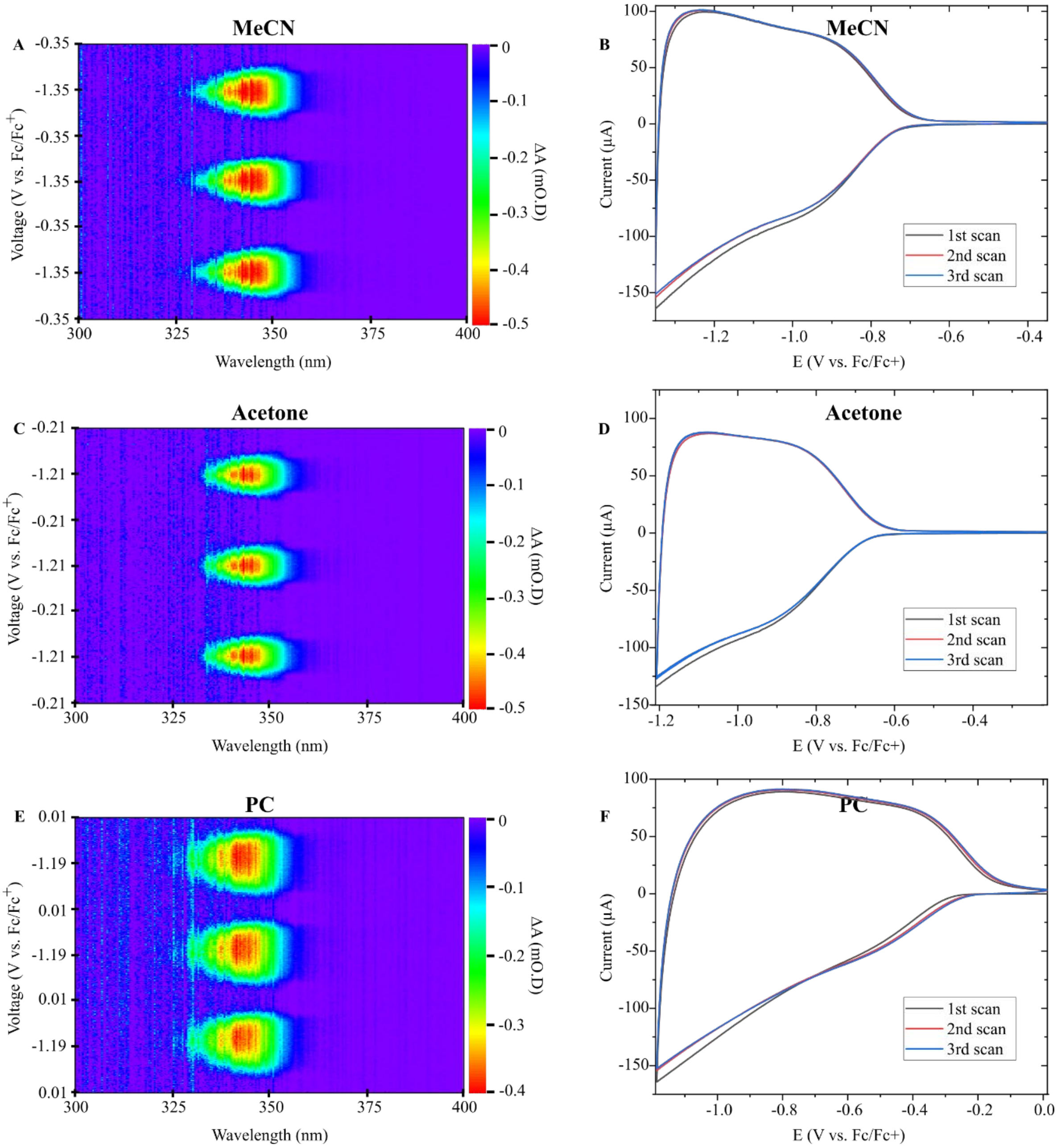


**Figure S19. A), C), E)** Voltage–wavelength–ΔA and **B), D), F)** cyclic voltammetry (50 mV/s) plots of ZnO cQDs solvated with different solvents. The solvent used is indicated above each plot. The measurements were performed with 0.1 M $LiClO_4$ as supporting electrolyte.

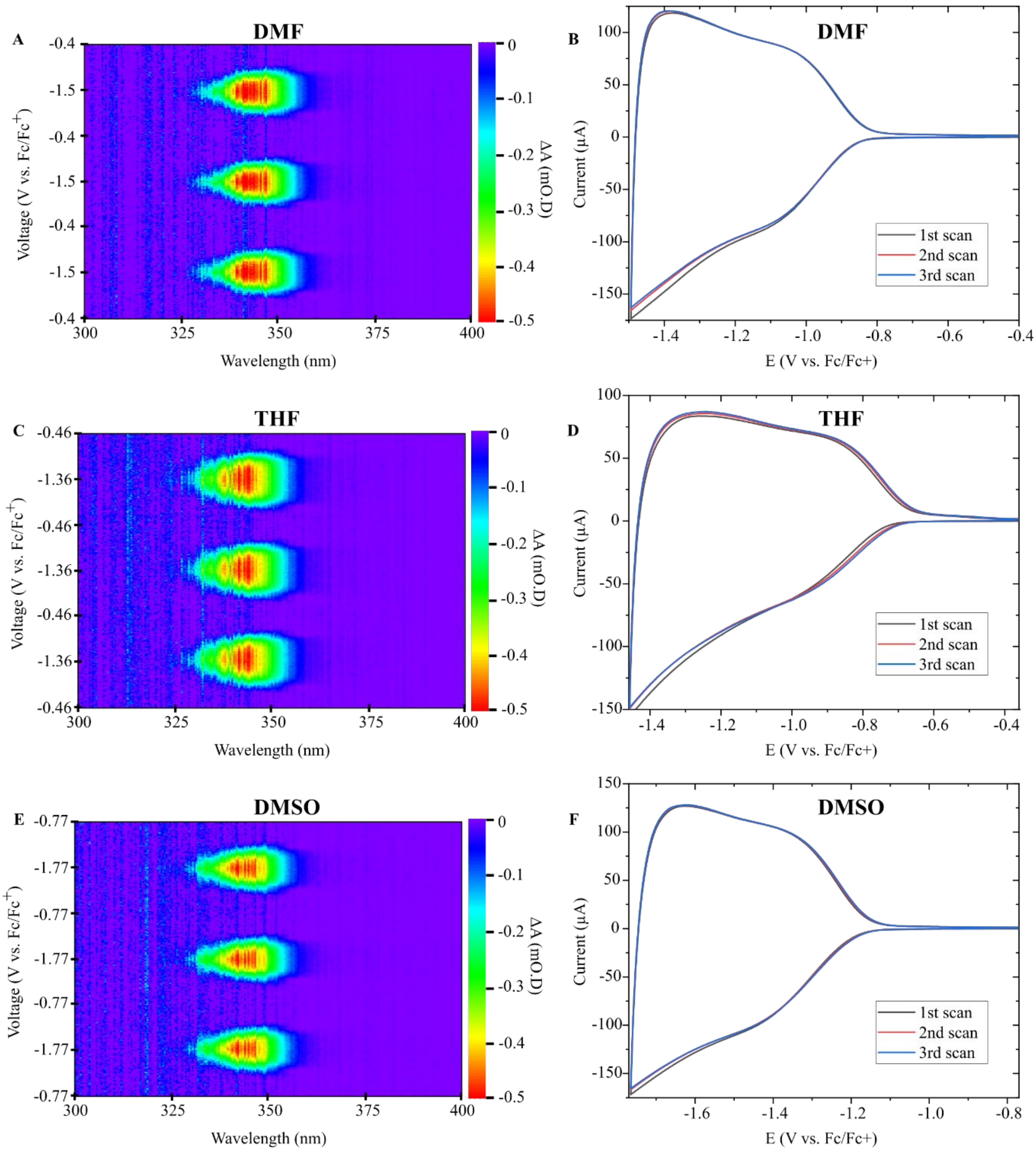


**Figure S20. A), C), E)** Voltage–wavelength–ΔA and **B), D), F)** cyclic voltammetry (50 mV/s) plots of ZnO cQDs solvated with different solvents. The solvent used is indicated above each plot. The measurements were performed with 0.1 M $LiClO_4$ as supporting electrolyte.

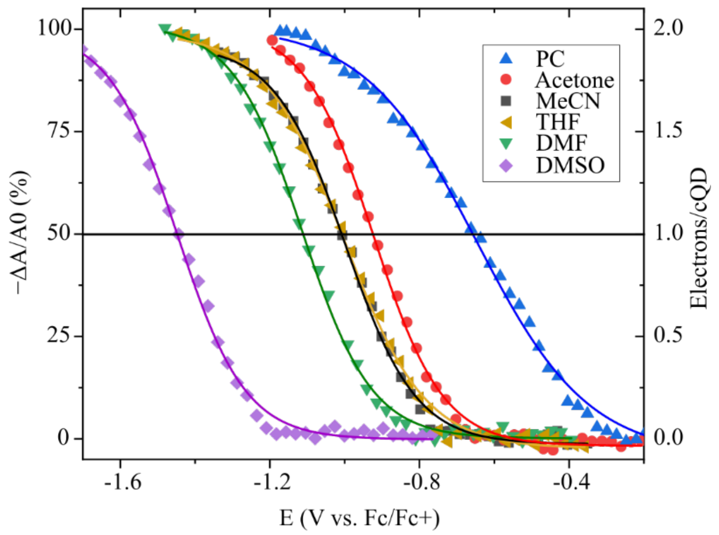


**Figure S21.** $\Delta A/A_0$–voltage plot of the $1S_h$-$1S_e$ transition for ZnO cQDs solvated with different solvents. The measurements were performed with 0.1 M $LiClO_4$ as supporting electrolyte. Lines are fits to a Boltzmann distribution function.

| Solvent | Bandgap (eV) |
|---|---|
| THF | 3.67 |
| PC | 3.67 |
| MeCN | 3.67 |
| DMSO | 3.67 |
| DMF | 3.67 |
| Acetone | 3.66 |

**Table S3. ZnO bandgap in different solvents.** The bandgap was measured from the position of the $1S_h$-$1S_e$ transition peak from the differential absorbance spectra for ZnO films immersed in different solvents.

## Effect of the cation in the conduction band energy level

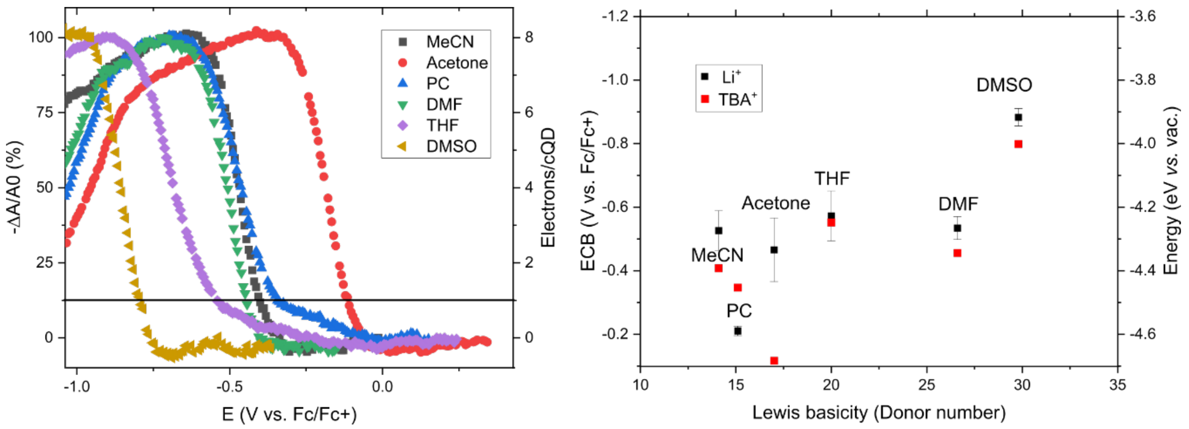


**Figure S22. A)** $\Delta A/A_0$–voltage plot of the $1S_h$-$1S_e$ transition for PbS-Br cQDs with a bandgap of 0.77 eV solvated with different solvents. The measurements were performed with 0.1 M $TBAClO_4$ as supporting electrolyte. **B)** Comparison of $E_{CB}$ values with Li+ and TBA+ cations as electrolyte.

# Computational calculations.

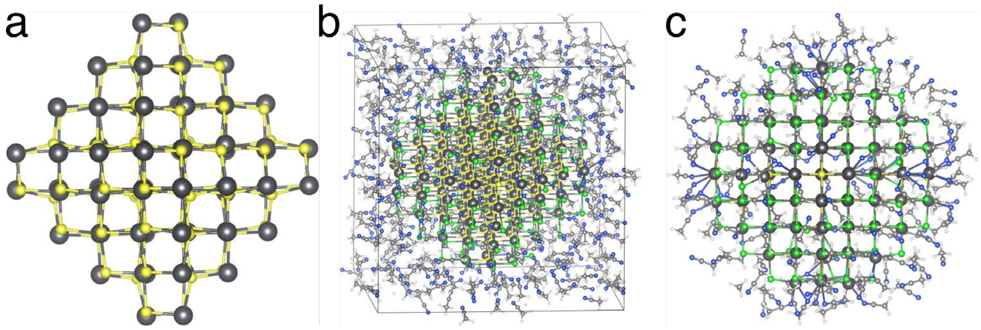


**Figure S23.** Three models constructed to simulate PbS quantum dot systems: **(a)** $Pb_{80}S_{80}$, **(b)** $Pb_{140}S_{85}Cl_{110}$ in an explicit solvent (345 acetonitrile molecules), and **(c)** $PbS_{140}S_{85}Cl_{110}$ surrounded by the first spherical layer of solvent molecules (156 acetonitrile molecules). Number of solvent molecules in a single layer vary from one solvent to the other (acetone: 127, DMF: 130, DMSO: 125, PC: 110, THF: 111).

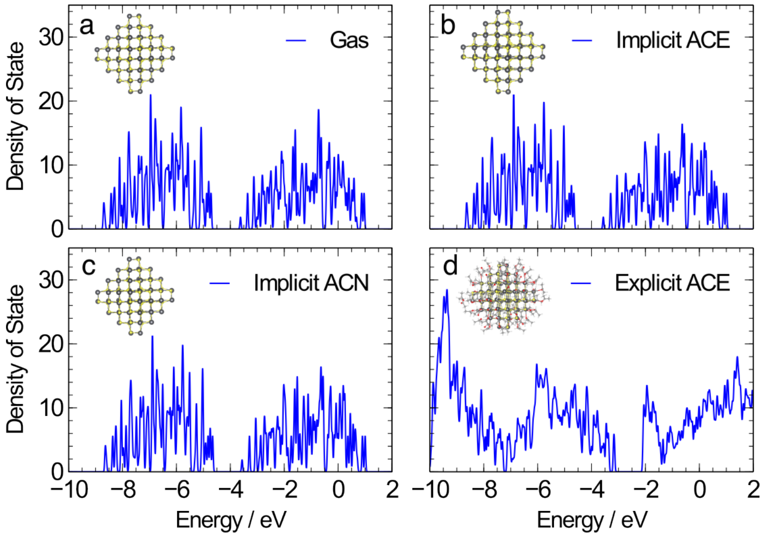


**Figure S24.** Density of states (DOSs) of a simplified $Pb_{80}S_{80}$ cQD in (a) vacuum, (b) implicit acetone, (c) implicit acetonitrile, and (d) explicit acetone formed with 150 acetone molecules. The DOS remain unchanged when comparing gas with implicit solvents, but changes drastically when explicit solvents are used. The simplified $Pb_{80}S_{80}$ model was used as a first test to explore the solvent influence.

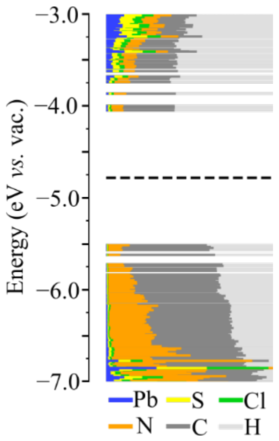


**Figure S25.** Density of states (DOSs) of the $Pb_{140}S_{85}Cl_{110}$ system in acetonitrile. Each horizontal line depicts a molecular orbital where the color indicates the fractional contribution to the molecular orbital of the corresponding element. The dashed black line indicates to which energy the molecular orbitals are filled with electrons. A DOS with a clean bandgap devoid of any trap states is obtained. The VB is found at -5.5 eV vs vacuum while the CB is found at -4.0 eV, giving a bandgap of 1.5 eV, which is reasonable given the very small size of the model.

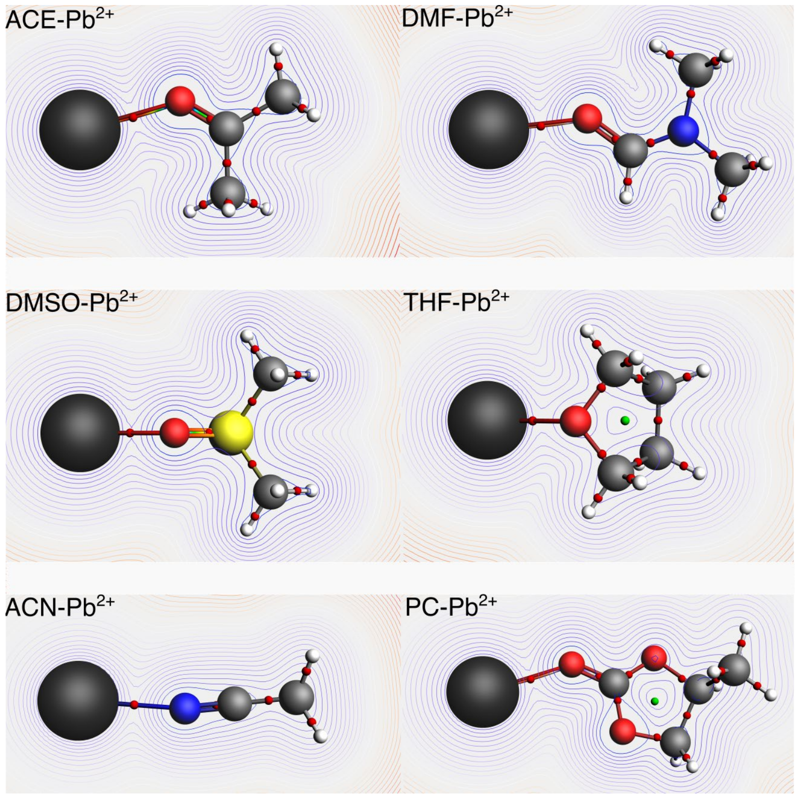


**Figure S26.** Quantum theory of atoms in molecules (QTAIM) analyses on model complexes between $Pb^{2+}$ and the various solvent molecule. Atoms are represented by balls (Pb: black, N: blue, C: grey, O: red, H: white, S: yellow). Red dots are bond critical points (BCP), and green dots are ring critical points. A cut-plane showing the electronic density contour for each simplified complex is provided. All complexes show a BCP between $Pb^{2+}$ and the coordinated atom of the solvent, which is indicative of a chemical bond between them.

| complex | BCP density $\rho_{cp}$ (a.u) | Laplacian $\nabla^2\rho_{cp}$ (a.u) | Total energy density $H_{cp}$ (a.u) | $E_{covalent}$ (kcal/mol) | $E_{non\text{-}covalent}$ (kcal/mol) | $E_{complexation}$ (kcal/mol) |
|---|---|---|---|---|---|---|
| $Pb^{2+}$-Acetone | 0.051 | 0.18 | -0.005 | -48.97 | -316.50 | -15.1 |
| $Pb^{2+}$-MeCN | 0.041 | 0.15 | -0.001 | -38.04 | -307.27 | -12.3 |
| $Pb^{2+}$-DMF | 0.059 | 0.24 | -0.005 | -57.77 | -341.16 | -20.2 |
| $Pb^{2+}$-DMSO | 0.064 | 0.28 | -0.006 | -64.18 | -363.28 | -21.8 |
| $Pb^{2+}$-PC | 0.045 | 0.17 | -0.002 | -44.24 | -321.61 | -10.6 |
| $Pb^{2+}$-THF | 0.054 | 0.20 | -0.006 | -51.22 | -315.35 | -19.4 |

**Table S4.** QTAIM calculations on model complexes between $Pb^{2+}$ and the various solvent molecules. All complexes show a bond critical point (BCP) between $Pb^{2+}$ and the coordinated atom of the solvent (see Figure S23). Shown here are the density ($\rho_{cp}$) at this BPC, the Laplacian of the electron density ($\nabla^2\rho_{cp}$), total energy density ($H_{cp}$) and the covalent ($E_{covalent}$) and non-covalent ($E_{non\text{-}covalent}$) contributions to the bonding interaction. Also shown is the counterpoise-corrected electronic energy for formation of the complex from the isolated $Pb^{2+}$ and solvent molecule ($E_{complexation}$). All calculations were performed at the wB97XD/ma-def2tzvp//wB97XD/def2sv(p) level of theory in their respective implicit solvents (see the methods section in the main text for details).

| solvent | Pb charge (e) | charge transfer amount (e) |
|---|---|---|
| Acetone | +1.937 | 0.063 |
| MeCN | +1.959 | 0.041 |
| DMF | +1.934 | 0.066 |
| DMSO | +1.934 | 0.066 |
| PC | +1.956 | 0.044 |
| THF | +1.947 | 0.053 |

**Table S5.** Amount of charge transfer (e) from a solvent molecule to $Pb^{2+}$ as calculated via Natural Bond Orbital (NBO) analyses at the wB97XD/ma-def2tzvp//wB97XD/def2sv(p) level of theory.

# Pseudoreference electrode calibration.

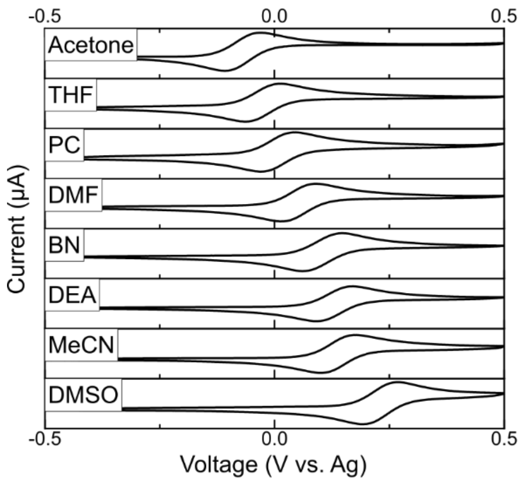


**Figure S27.** Cyclic voltammograms of ferrocenium ($10^{-3}$ mol $L^{-1}$) in $LiClO_4$ (0.1 mol $L^{-1}$) in different solvents used to calibrate the $Ag/Ag^{+}$? pseudoreference electrode. The measurements were performed with a polycrystalline gold as working electrode, a silver wire as pseudoreference electrode and a platinum coil as counter electrode.

# Bond critical point (BCP) data

Acetone-$Pb^{2+}$:
CP # 15
(RANK,SIGNATURE): (3,-1)

CP COORDINATES: 0.230297 0.299522 -0.014066

EIGENVALUES OF HESSIAN MATRIX:

-0.5536566E-01 -0.5524654E-01 0.2951543E+00

EIGENVECTORS (ORTHONORMAL) OF HESSIAN MATRIX (COLUMNS):

0.7359708E-01 0.2422903E+00 -0.9674083E+00
-0.2353277E+00 -0.9384268E+00 -0.2529347E+00
0.9691255E+00 -0.2462733E+00 0.1204784E-01

HESSIAN MATRIX:

0.2726856E+00 0.8574206E-01 -0.4092480E-02
-0.3283590E-01 -0.1040617E-02
-0.5530756E-01

VALUES OF SOME FUNCTIONS AT CPs (a.u.):

Rho = 0.5101866E-01
|GRAD(Rho)| = 0.1500656E-12
GRAD(Rho)x = 0.1488438E-12
GRAD(Rho)y = 0.1911072E-13
GRAD(Rho)z = 0.4828934E-16
Laplacian = 0.1845421E+00
(-1/4)Del**2(Rho)) = -0.4613553E-01
Diamond = 0.9323361E-01
Metallicity = 0.2620592E+01 Comput.Theor.Chem. 1053 (2015) 112
Ellipticity = 0.2156026E-02

INHOMOGENEITY PARAMETERS AT CPs (a.u.)

Delta_u = 0.1600177E+00 J.Phys.Chem.A, 113 (2009) 12322; PCCP, 16 (2014) 14539
Delta_s = 0.1366761E+01 >>
Delta_t = 0.1297465E+01 >>
Inhom_s = 0.7316571E+00 Chem.Phys.Lett. 579 (2013) 122
Inhom_ex = 0.9605981E+00 >>

ENERGY COMPONENTS AT CPs (a.u.)

Gb(Abramov) = 0.5090742E-01 ActaCryst. 153 (1997) 264; Chem.Phys.Lett. 285 (1998) 170
Vb(Abramov) = -0.5567931E-01 >>
Hb(Abramov) = -0.4771888E-02 >>
Ex_ueg = -0.1397517E-01 PCCP, 16 (2014) 14539
Ec_ueg = -0.2471258E-02 >>
Delta_ex = -0.1514515E-01 >>
Delta_ec = 0.5279334E-02 >>
Delta_et = -0.5589700E-01 >>
Pxc = 0.5655082E+01 JCP, 132 (2010) 211101
Qxc = -0.2868762E+01 >>

-------------------------------------------------------

Acetonitrile-$Pb^{2+}$:

CP # 13

(RANK,SIGNATURE): (3,-1)

CP COORDINATES: 0.469217 -0.087014 -0.000316

EIGENVALUES OF HESSIAN MATRIX:

-0.4129285E-01 -0.4126470E-01 0.2361195E+00

EIGENVECTORS (ORTHONORMAL) OF HESSIAN MATRIX (COLUMNS):

0.2253307E-02 0.7852035E-01 -0.9969100E+00
0.3215057E-01 0.9963914E+00 0.7855218E-01
-0.9994805E+00 0.3222822E-01 0.2792984E-03

HESSIAN MATRIX:

0.2344079E+00 -0.2172181E-01 -0.7717019E-04
-0.3955315E-01 0.6990204E-05
-0.4129280E-01

VALUES OF SOME FUNCTIONS AT CPs (a.u.):

Rho = 0.4129341E-01
|GRAD(Rho)| = 0.3731811E-16
GRAD(Rho)x = 0.3715007E-16
GRAD(Rho)y = -0.3537334E-17
GRAD(Rho)z = -0.1798018E-19
Laplacian = 0.1535620E+00
(-1/4)Del**2(Rho)) = -0.3839050E-01
Diamond = 0.5916031E-01
Metallicity = 0.2213760E+01 Comput.Theor.Chem. 1053 (2015) 112
Ellipticity = 0.6821543E-03

INHOMOGENEITY PARAMETERS AT CPs (a.u.)

Delta_u = 0.1269607E+00 J.Phys.Chem.A, 113 (2009) 12322; PCCP, 16 (2014) 14539
Delta_s = 0.1437666E+01 >>
Delta_t = 0.1317508E+01 >>
Inhom_s = 0.6955718E+00 Chem.Phys.Lett. 579 (2013) 122
Inhom_ex = 0.9371705E+00 >>

ENERGY COMPONENTS AT CPs (a.u.)

Gb(Abramov) = 0.3975823E-01 ActaCryst. 153 (1997) 264; Chem.Phys.Lett. 285 (1998) 170
Vb(Abramov) = -0.4112595E-01 >>
Hb(Abramov) = -0.1367728E-02 >>
Ex_ueg = -0.1054126E-01 PCCP, 16 (2014) 14539
Ec_ueg = -0.1939583E-02 >>
Delta_ex = -0.1200205E-01 >>
Delta_ec = 0.4496798E-02 >>
Delta_et = -0.4506308E-01 >>
Pxc = 0.5434809E+01 JCP, 132 (2010) 211101
Qxc = -0.2669021E+01 >>

-------------------------------------------------------

DMF-$Pb^{2+}$:

CP #   20
(RANK,SIGNATURE):     (3,-1)

CP COORDINATES:     -0.179012     0.067862     0.000050

EIGENVALUES OF HESSIAN MATRIX:

-0.6597645E-01  -0.6451005E-01   0.3745970E+00

EIGENVECTORS (ORTHONORMAL) OF HESSIAN MATRIX (COLUMNS):

0.8642238E-01  -0.7726905E-05   0.9962586E+00
-0.9962584E+00  -0.5546796E-03   0.8642236E-01
-0.5519365E-03   0.9999998E+00   0.5563472E-04

HESSIAN MATRIX:

0.3713065E+00   0.3793294E-01   0.2440814E-04
-0.6268588E-01   0.1304933E-05
-0.6451005E-01

VALUES OF SOME FUNCTIONS AT CPs (a.u.):

Rho =    0.5856857E-01
|GRAD(Rho)| =    0.6834184E-13
GRAD(Rho)x =   -0.6831909E-13
GRAD(Rho)y =    0.1749997E-14
GRAD(Rho)z =    0.2176132E-15
Laplacian =    0.2441105E+00
(-1/4)Del**2(Rho)) =   -0.6102763E-01
Diamond =    0.1488374E+00
Metallicity =    0.2493452E+01     Comput.Theor.Chem. 1053 (2015) 112
Ellipticity =    0.2273131E-01

INHOMOGENEITY PARAMETERS AT CPs (a.u.)

Delta_u =    0.2013357E+00     J.Phys.Chem.A, 113 (2009) 12322; PCCP, 16 (2014) 14539
Delta_s =    0.1430642E+01     >>
Delta_t =    0.1389708E+01     >>
Inhom_s =    0.6989868E+00     Chem.Phys.Lett. 579 (2013) 122
Inhom_ex =    0.8996709E+00     >>

ENERGY COMPONENTS AT CPs (a.u.)

Gb(Abramov) =    0.6604667E-01     ActaCryst. 153 (1997) 264; Chem.Phys.Lett. 285 (1998) 170
Vb(Abramov) =   -0.7106570E-01     >>
Hb(Abramov) =   -0.5019036E-02     >>
Ex_ueg =   -0.1679853E-01     PCCP, 16 (2014) 14539
Ec_ueg =   -0.2893716E-02     >>
Delta_ex =   -0.2075410E-01     >>
Delta_ec =    0.6978319E-02     >>
Delta_et =   -0.7652273E-01     >>
Pxc =    0.5805175E+01     JCP, 132 (2010) 211101
Qxc =   -0.2974083E+01     >>

---------------------------------------------------------

DMSO-$Pb^{2+}$:
CP #   15

(RANK,SIGNATURE): (3,-1)

CP COORDINATES: -0.080331 0.000103 0.125696

EIGENVALUES OF HESSIAN MATRIX:

-0.7263512E-01 -0.6988553E-01 0.4263553E+00

EIGENVECTORS (ORTHONORMAL) OF HESSIAN MATRIX (COLUMNS):

-0.1205666E+00 -0.4579687E-04 -0.9927052E+00
0.2772192E-03 0.1000000E+01 -0.7980239E-04
0.9927052E+00 -0.2848185E-03 -0.1205666E+00

HESSIAN MATRIX:

0.4191018E+00 0.3940422E-04 0.5972271E-01
-0.6988553E-01 0.4017904E-05
-0.6538165E-01

VALUES OF SOME FUNCTIONS AT CPs (a.u.):

Rho = 0.6374133E-01
|GRAD(Rho)| = 0.3271928E-16
GRAD(Rho)x = -0.3253848E-16
GRAD(Rho)y = 0.2373261E-19
GRAD(Rho)z = -0.3434791E-17
Laplacian = 0.2838347E+00
(-1/4)Del**2(Rho)) = -0.7095866E-01
Diamond = 0.1919387E+00
Metallicity = 0.2469351E+01 Comput.Theor.Chem. 1053 (2015) 112
Ellipticity = 0.3934415E-01

INHOMOGENEITY PARAMETERS AT CPs (a.u.)

Delta_u = 0.2281272E+00 J.Phys.Chem.A, 113 (2009) 12322; PCCP, 16 (2014) 14539
Delta_s = 0.1448033E+01 >>
Delta_t = 0.1426584E+01 >>
Inhom_s = 0.6905918E+00 Chem.Phys.Lett. 579 (2013) 122
Inhom_ex = 0.8744454E+00 >>

ENERGY COMPONENTS AT CPs (a.u.)

Gb(Abramov) = 0.7650942E-01 ActaCryst. 153 (1997) 264; Chem.Phys.Lett. 285 (1998) 170
Vb(Abramov) = -0.8206018E-01 >>
Hb(Abramov) = -0.5550759E-02 >>
Ex_ueg = -0.1880528E-01 PCCP, 16 (2014) 14539
Ec_ueg = -0.3187452E-02 >>
Delta_ex = -0.2459317E-01 >>
Delta_ec = 0.7983896E-02 >>
Delta_et = -0.8934461E-01 >>
Pxc = 0.5899785E+01 JCP, 132 (2010) 211101
Qxc = -0.3080347E+01 >>

---------------------------------------------------------

PC-$Pb^{2+}$:
CP # 17
(RANK,SIGNATURE): (3,-1)

CP COORDINATES: 0.572580 -0.317828 -0.145740

EIGENVALUES OF HESSIAN MATRIX:

-0.4793570E-01 -0.4742553E-01 0.2680624E+00

EIGENVECTORS (ORTHONORMAL) OF HESSIAN MATRIX (COLUMNS):

0.3089906E+00 -0.7712658E-01 0.9479326E+00
-0.9143397E+00 -0.2983907E+00 0.2737626E+00
-0.2617399E+00 0.9513225E+00 0.1627198E+00

HESSIAN MATRIX:

0.2360157E+00 0.8201593E-01 0.4870446E-01
-0.2420750E-01 0.1393182E-01
-0.3910708E-01

VALUES OF SOME FUNCTIONS AT CPs (a.u.):

Rho = 0.4543889E-01
|GRAD(Rho)| = 0.1460897E-15
GRAD(Rho)x = -0.1399416E-15
GRAD(Rho)y = -0.3816439E-16
GRAD(Rho)z = -0.1737927E-16
Laplacian = 0.1727011E+00
(-1/4)Del**2(Rho)) = -0.4317528E-01
Diamond = 0.7640445E-01
Metallicity = 0.2308682E+01 Comput.Theor.Chem. 1053 (2015) 112
Ellipticity = 0.1075720E-01

INHOMOGENEITY PARAMETERS AT CPs (a.u.)

Delta_u = 0.1444222E+00 J.Phys.Chem.A, 113 (2009) 12322; PCCP, 16 (2014) 14539
Delta_s = 0.1439550E+01 >>
Delta_t = 0.1340437E+01 >>
Inhom_s = 0.6946615E+00 Chem.Phys.Lett. 579 (2013) 122
Inhom_ex = 0.9296804E+00 >>

ENERGY COMPONENTS AT CPs (a.u.)

Gb(Abramov) = 0.4539652E-01 ActaCryst. 153 (1997) 264; Chem.Phys.Lett. 285 (1998) 170
Vb(Abramov) = -0.4761776E-01 >>
Hb(Abramov) = -0.2221238E-02 >>
Ex_ueg = -0.1197536E-01 PCCP, 16 (2014) 14539
Ec_ueg = -0.2164348E-02 >>
Delta_ex = -0.1385547E-01 >>
Delta_ec = 0.5108598E-02 >>
Delta_et = -0.5252221E-01 >>
Pxc = 0.5533011E+01 JCP, 132 (2010) 211101
Qxc = -0.2712187E+01 >>

-------------------------------------------------------
PC-$Pb^{2+}$:
CP # 15
(RANK,SIGNATURE): (3,-1)

CP COORDINATES: 0.090274 -0.000237 0.331273

EIGENVALUES OF HESSIAN MATRIX:

-0.6238581E-01 -0.5552423E-01 0.3168862E+00

EIGENVECTORS (ORTHONORMAL) OF HESSIAN MATRIX (COLUMNS):

-0.6917221E-02 -0.3258574E+00 0.9453936E+00
-0.9997425E+00 0.2268709E-01 0.5048868E-03
0.2161275E-01 0.9451467E+00 0.3259304E+00

HESSIAN MATRIX:

0.2773244E+00 0.1303069E-03 0.1147528E+00
-0.6238218E-01 0.2095425E-03
-0.1596604E-01

VALUES OF SOME FUNCTIONS AT CPs (a.u.):

Rho = 0.5411096E-01
|GRAD(Rho)| = 0.3093725E-14
GRAD(Rho)x = 0.2942267E-14
GRAD(Rho)y = 0.1845802E-19
GRAD(Rho)z = 0.9561373E-15
Laplacian = 0.1989762E+00
(-1/4)Del**2(Rho)) = -0.4974404E-01
Diamond = 0.1073918E+00
Metallicity = 0.2680942E+01 Comput.Theor.Chem. 1053 (2015) 112
Ellipticity = 0.1235781E+00

INHOMOGENEITY PARAMETERS AT CPs (a.u.)

Delta_u = 0.1716746E+00 J.Phys.Chem.A, 113 (2009) 12322; PCCP, 16 (2014) 14539
Delta_s = 0.1355674E+01 >>
Delta_t = 0.1299625E+01 >>
Inhom_s = 0.7376403E+00 Chem.Phys.Lett. 579 (2013) 122
Inhom_ex = 0.9566234E+00 >>

ENERGY COMPONENTS AT CPs (a.u.)

Gb(Abramov) = 0.5538951E-01 ActaCryst. 153 (1997) 264; Chem.Phys.Lett. 285 (1998) 170
Vb(Abramov) = -0.6103498E-01 >>
Hb(Abramov) = -0.5645471E-02 >>
Ex_ueg = -0.1511583E-01 PCCP, 16 (2014) 14539
Ec_ueg = -0.2643341E-02 >>
Delta_ex = -0.1651772E-01 >>
Delta_ec = 0.5584922E-02 >>
Delta_et = -0.5999073E-01 >>
Pxc = 0.5718456E+01 JCP, 132 (2010) 211101
Qxc = -0.2957556E+01 >>

---------------------------------------------------------
C-C BCP in acetonitrile:
CP # 12
(RANK,SIGNATURE): (3,-1)

CP COORDINATES: 3.594294 0.018129 -0.000331

EIGENVALUES OF HESSIAN MATRIX:

-0.5019672E+00 -0.5008784E+00 0.3470319E+00

EIGENVECTORS (ORTHONORMAL) OF HESSIAN MATRIX (COLUMNS):

| | | |
|---|---|---|
| 0.1448989E-01 | 0.9989810E-01 | 0.9948922E+00 |
| -0.1466673E+00 | -0.9840220E+00 | 0.1009427E+00 |
| 0.9890798E+00 | -0.1473808E+00 | 0.3934074E-03 |

HESSIAN MATRIX:

| | | |
|---|---|---|
| 0.3383919E+00 | 0.8515551E-01 | 0.3162655E-03 |
| | -0.4922621E+00 | 0.1916243E-03 |
| | | -0.5019435E+00 |

VALUES OF SOME FUNCTIONS AT CPs (a.u.):

Rho = 0.2637767E+00
|GRAD(Rho)| = 0.2087163E-12
GRAD(Rho)x = -0.2073754E-12
GRAD(Rho)y = -0.2357297E-13
GRAD(Rho)z = -0.1498332E-14
Laplacian = -0.6558137E+00
(-1/4)Del**2(Rho)) = 0.1639534E+00
Diamond = 0.6232815E+00
Metallicity = -0.1139970E+02 Comput.Theor.Chem. 1053 (2015) 112
Ellipticity = 0.2173850E-02

INHOMOGENEITY PARAMETERS AT CPs (a.u.)

Delta_u = 0.4536063E+00 J.Phys.Chem.A, 113 (2009) 12322; PCCP, 16 (2014) 14539
Delta_s = 0.4333713E+00 >>
Delta_t = 0.5409801E+00 >>
Inhom_s = 0.2307490E+01 Chem.Phys.Lett. 579 (2013) 122
Inhom_ex = 0.1374790E+01 >>

ENERGY COMPONENTS AT CPs (a.u.)

Gb(Abramov) = 0.2022010E+00 ActaCryst. 153 (1997) 264; Chem.Phys.Lett. 285 (1998) 170
Vb(Abramov) = -0.5683555E+00 >>
Hb(Abramov) = -0.3661545E+00 >>
Ex_ueg = -0.1249397E+00 PCCP, 16 (2014) 14539
Ec_ueg = -0.1609820E-01 >>
Delta_ex = 0.6610395E-01 >>
Delta_ec = 0.1292050E-01 >>
Delta_et = -0.3135863E+00 >>
Pxc = 0.7761095E+01 JCP, 132 (2010) 211101
Qxc = 0.5116205E+01 >>
